# Loss of PV Interneurons in the BLA May Contribute to Altered Network and Behavioral States in Chronically Epileptic Mice

**DOI:** 10.1523/ENEURO.0482-23.2024

**Published:** 2025-01-16

**Authors:** Phillip L.W. Colmers, Pantelis Antonoudiou, Trina Basu, Emanuel M. Coleman, Yingchu He, Garrett Scapa, Patrick Fuller, Jamie Maguire

**Affiliations:** ^1^Department of Neuroscience, Tufts University School of Medicine, Boston, Massachusetts 02111; ^2^Department of Neurological Surgery, UC Davis Health, Sacramento, California 95817

**Keywords:** anxiety, basolateral amygdala, depression, epilepsy, interneurons, local field potentials, network states, oscillations, psychiatric comorbidities

## Abstract

Psychiatric disorders, including anxiety and depression, are highly comorbid in people with epilepsy. However, the mechanisms mediating the shared pathophysiology are currently unknown. There is considerable evidence implicating the basolateral amygdala (BLA) in the network communication of anxiety and fear, a process demonstrated to involve parvalbumin-positive (PV) interneurons. The loss of PV interneurons has been well described in the hippocampus of chronically epileptic mice and in postmortem human tissue of patients with temporal lobe epilepsy (TLE). We hypothesize that a loss of PV interneurons in the BLA may contribute to comorbid mood disorders in epilepsy. To test this hypothesis, we employed a ventral intrahippocampal kainic acid model of TLE in mice, which exhibits profound behavioral deficits associated with chronic epilepsy. We demonstrate a loss of PV interneurons and dysfunction of the remaining PV interneurons in the BLA of chronically epileptic mice. Furthermore, we demonstrate altered principal neuron function and impaired coordination of BLA network and behavioral states in chronically epileptic mice. To determine whether the loss of PV interneurons contributes to these altered network and behavioral states, we partially ablated PV interneurons in the BLA by stereotaxically injecting AAV-Flex-DTA into the BLA of PV-Cre mice. Loss of PV interneurons in the BLA is sufficient to alter behavioral states, such as increasing avoidance behaviors and impairing fear learning. These data suggest that compromised inhibition in the BLA in chronically epileptic mice may contribute to behavioral deficits, suggesting a novel mechanism contributing to comorbid anxiety and epilepsy.

## Significance Statement

Psychiatric illnesses and epilepsy are highly comorbid and negatively impact the quality of life of people with epilepsy. The pathophysiological mechanisms mediating the bidirectional relationship between mood disorders and epilepsy remain unknown, and, therefore, treatment options remain inadequate. Here we demonstrate a potential novel mechanism, involving the loss of parvalbumin-positive interneurons in the basolateral amygdala, leading to a corruption of network and behavioral states in mice. These findings pinpoint a critical node and demonstrate a potential novel cellular and circuit mechanism involved in the comorbidity of psychiatric illnesses and epilepsy.

## Introduction

Psychiatric illnesses are highly comorbid with epilepsy, occurring in up to 75% of people with epilepsy (LaFrance et al., 2008). Depression and anxiety are the most prevalent psychiatric comorbidities in epilepsy, with an incidence of 55 and 25–50%, respectively (Lambert and Robertson, 1999; Brandt et al., 2010). Thus, the incidence is much higher than the general population or in patients with other chronic illnesses (Josephson and Jetté, 2017). Evidence suggests that there is a bidirectional relationship between psychiatric disorders and epilepsy and is thought to involve an unknown shared neurobiological mechanism (Kanner and Balabanov, 2002; Mula, 2012; Kanner et al., 2018). Behavioral deficits associated with chronic epilepsy have also been demonstrated in animal models (Zeidler et al., 2018), including increased avoidance behaviors and anhedonia. However, the neurobiological mechanisms mediating the behavioral deficits associated with chronic epilepsy are not fully understood. The goal of the current study is to investigate a novel potential mechanism contributing to these common comorbidities and potentially mediating the bidirectional relationship between mood disorders and epilepsy.

The amygdala plays an integral role in emotional processing (LeDoux, 1992) and has been implicated in the pathophysiology of mood disorders (Price and Drevets, 2009; Ressler, 2010). The neural computational functions of the amygdala are subserved in part by oscillations, and extensive evidence demonstrates the role of specific oscillatory states within and between the mPFC and basolateral amygdala (BLA) in governing behavioral outcomes (Likhtik et al., 2014; Stujenske et al., 2014; Felix-Ortiz et al., 2016; Davis et al., 2017; Ozawa et al., 2020; for review, see Tovote et al., 2015; Antonoudiou et al., 2022). Thus, disruptions in the ability to orchestrate appropriate neural oscillations within and between the mPFC and BLA are likely to result in deficits in emotional processing.

Oscillations have been shown to be generated by neuronal synchrony that is orchestrated by GABAergic interneurons (Buzsáki and Wang, 2012). In particular, parvalbumin-positive (PV) interneurons have been shown to be critical for the generation of gamma oscillations (Bartos et al., 2007). In the BLA, oscillations are driven by GABAergic signaling, and PV interneurons play a critical role in generating oscillations in this network (Antonoudiou et al., 2022). Furthermore, PV interneurons have been shown to be required for the network communication of fear (Davis et al., 2017). Thus, it is well established that PV interneurons in the BLA play a critical role in generating network and behavioral states.

Loss of GABAergic interneurons has been demonstrated both in experimental models of epilepsy and in people with epilepsy, particularly in the hippocampus (Liu et al., 2014). A loss of interneurons has also been demonstrated in the amygdala both in patients with temporal lobe epilepsy (TLE) and in experimental epilepsy models (Callahan et al., 1991; Tuunanen et al., 1996; Pitkanen et al., 1998; nicely reviewed in Aroniadou-Anderjaska et al., 2008). In particular, a loss of somatostatin-positive interneurons in the amygdala has been demonstrated in experimental epilepsy models (Sperk et al., 1986; Tuunanen et al., 1997). However, there is a discrepancy between the magnitude of the loss of GABA immunoreactive interneurons and SST interneurons (Pitkanen et al., 1998), suggesting that other subclasses of interneurons may be affected. Although there is extensive evidence for a loss and unique vulnerability of PV interneurons in the hippocampus of human TLE and in experimental models of epilepsy (Bouilleret et al., 2000; Marx et al., 2013; Houser, 2014), surprisingly few studies have investigated the potential loss of PV interneurons in the amygdala. PV interneurons make up nearly 50% of GABAergic interneurons in the rodent BLA (Rainnie et al., 2006; Sosulina et al., 2006; McDonald et al., 2012; for review see Capogna, 2014), whereas SST interneurons only represent ∼5% of this population (Rainnie et al., 2006). Here we demonstrate a loss of PV interneurons in the BLA of chronically epileptic mice, consistent with limited clinical findings suggesting abnormalities in PV interneurons in patients with TLE (D. M. Yilmazer-Hanke et al., 2007). It is likely that interneuron deficits in the BLA contribute to network and behavioral changes associated with chronic epilepsy; however, given the demonstrated importance of PV interneurons in the BLA for orchestrating network and behavioral states, this study focused on the loss of PV interneurons in the BLA and the impact on comorbid behavioral deficits in chronic epilepsy.

The current study tests the hypothesis that the loss of PV interneurons in the BLA corrupts oscillatory states in the BLA, contributing to a breakdown in the network coordination of behavioral states and increasing behavioral deficits in chronically epileptic mice. Here we demonstrate that chronically epileptic mice exhibit profound behavioral deficits, consistent with previous reports (Zeidler et al., 2018), including increased avoidance behaviors, anhedonia, and impaired fear learning. Chronically epileptic mice exhibit a substantial reduction in the number of PV interneurons in the BLA and altered inhibitory signaling. The reduction in the number and dysfunction of PV interneurons in the BLA is associated with altered network activity in the BLA. Partial ablation of PV interneurons in the BLA is sufficient to induce behavioral deficits in nonepileptic mice. These data suggest that degeneration of PV interneurons in the BLA of chronically epileptic mice and disruption in the ability to coordinate network states may contribute to comorbid behavioral deficits.

## Materials and Methods

### Animals

Adult male C57BL/6J (stock #000664), PV-Cre (stock #017320), and PV-tdTomato (stock #027395) mice, aged 10–12 weeks old, were purchased from The Jackson Laboratory and group housed (four/cage) in temperature- and humidity-controlled housing rooms on a 12 h light/dark cycle (lights on at 7A.M.) with *ad libitum* food and water. Animals were handled according to protocols and procedures approved by the Tufts University Institutional Animal Care and Use Committee. All chronically epileptic mice had verified spontaneous recurrent seizures (SRS) in the ventral intrahippocampal kainic acid (vIHKA) group.

### Behavior paradigms

#### Open field

Avoidance behaviors in the open-field test were measured as previously described (Antonoudiou et al., 2022; Walton et al., 2023; Basu et al., 2024). Briefly, mice were individually placed into the center of the open arena (40 × 40 cm) equipped with a photobeam frame with 16 × 16 equally spaced photocells (Hamilton-Kinder). The number of beam breaks, entries, time spent, and distance traveled in the center and the periphery of the apparatus were automatically measured using the Motor Monitor software (Hamilton-Kinder) over the 10 min testing period.

#### Light/dark box

Avoidance behaviors in the light/dark box were measured as previously described (Melón et al., 2018; Antonoudiou et al., 2022; Walton et al., 2023; Basu et al., 2024). Briefly, mice were placed individually into the dark compartment of the two-chamber light/dark box apparatus (22 × 43 cm) equipped with a photobeam frame with eight equally spaced photocells (Hamilton-Kinder). Beam breaks, number of entries, time spent, and distance traveled in the light and dark compartments were automatically measured using the Motor Monitor software (Hamilton-Kinder) during the 10 min testing period.

#### Elevated plus maze

The elevated plus maze test was conducted as previously described (Melón et al., 2018; Antonoudiou et al., 2022; Walton et al., 2023). Briefly, mice were individually placed into the center of the elevated plus maze which consists of two opposing 38 × 6.5-cm-wide arms, standing 75 cm from the ground, one arm exposed and one with closed walls (10 cm high). All arms of the elevated plus maze. Movement was captured either using 48 equally spaced photocells (Motor Monitor software, Hamilton-Kinder, or EthoVision software, Noldus), enabling automated measurements of beam breaks, number of entries, distance traveled, and total time spent in the open and closed arms during the 10 min test.

#### Sucrose preference

Anhedonia was measured as previously described by our laboratory (Antonoudiou et al., 2022; Basu et al., 2024). Mice were individually housed and given *ad libitum* access to two water bottles, one filled with water and the other filled with 2% sucrose (*w*/*v*), for 7 consecutive days. The positions of the water bottles were alternated daily to avoid placement preference. The amount of water and sucrose solution consumed was measured daily, and sucrose preference was measured as the percentage of sucrose consumed compared with total volume consumed.

#### Fear conditioning

Fear conditioning was performed as previously described (Davis et al., 2017; Ozawa et al., 2020). Mice were individually placed into the fear conditioning chambers (Coulbourn Instruments; H10-11R-TC, 12″W × 10″D × 12″H) and were subjected to a series of three 20 s tones (2,800 Hz, 80 dB) which ended simultaneously with footshocks (2 s, 0.7 mA) separated by a 1 min interval over a 6 min testing period. Twenty-five hours later, the recall of the contextual fear memory was measured by returning the mice to the fear conditioning chambers for a 3 min period, and the amount of freezing behavior was measured in the absence of the tone or shock (contextual recall). Three hours later, cued fear conditioning was measured in a novel environment, a rectangular plastic container with black and white stripes along the sides and bedding scented with 1% acetic acid, in response to presentation with the same tone protocol that was presented during the training phase (3 min baseline and three 20 s tones separated by a 60 s gap) without shocks (cued recall). Freezing behavior was analyzed using the Actimetrics FreezeFrame software (Coulbourn Instruments, bout length 1 s). The percentage time freezing for 40 s after each tone was calculated as a measure of cued fear memory. The freezing behavior during the training phase was also measured to assess any potential baseline differences in freezing behavior unrelated to memory.

### Immunohistochemistry

Immunohistochemistry was performed as previously described (Hooper and Maguire, 2016; Melón et al., 2018; DiLeo et al., 2022). Mice were anesthetized with isoflurane and decapitated, and the brain was rapidly extracted. The brain was fixed by immersion fixation overnight in 4% PFA at 4°C and then cryoprotected in 10–30% sucrose. The brains were then flash frozen in isopentane on dry ice and stored at −80°C until cryosectioning. Free-floating 40 μm coronal slices were incubated in a monoclonal antibody against parvalbumin (PV; 1:1,000, Sigma-Aldrich P3088) for 24 h at 4°C followed by incubation with a biotinylated goat anti-mouse (1:200, Thermo Fisher Scientific A28181) antibody for 2 h at room temperature and then a streptavidin-conjugated Alexa Fluor 488 (1:200, Thermo Fisher Scientific S32354) for 2 h at room temperature. Slices were mounted and coverslipped with an antifade hard set mounting medium with DAPI (VECTASHIELD H1500). Fluorescence imaging was performed using a Nikon A1R confocal microscope and the number of PV-positive cells in the BLA was measured bilaterally in both the vIHKA and AAV-DTA models versus respective controls using the ImageJ software.

### Electrophysiology

Mice were anesthetized with isoflurane and rapidly decapitated, and the brain was rapidly extracted and placed in an ice-cold slicing solution containing (in mM) 150 sucrose, 15 glucose, 33 NaCl, 25 NaHCO_3_, 2.5 KCl, 1.25 NaH_2_PO_4_, 1 CaCl_2_, 7 MgCl_2_ (300–310 mOsm). Coronal sections (350 μm) were prepared on a vibratome and incubated at 33°C in normal artificial cerebral spinal fluid (aCSF) containing the following for at least 1 h prior to recording (in mM): 126 NaCl, 10 glucose, 2 MgCl_2_, 2 CaCl_2_, 2.5 KCl, 1.25 NaHCO_3_, 1.5 Na-pyruvate, and 1 L-glutamine (300–310 mOsm). Electrophysiological measurements were performed at 33°C (maintained using in line heater, Warner Instruments) and continuously perfused with aCSF bubbled with 95% O2% and 5% CO2 at a rate of ≥4 ml/min. Voltage-clamp and current-clamp recordings were performed on visually identified principal neurons in the BLA and fluorescently labeled PV interneurons (PV-tdTomato) using a 200B Axopatch amplifier (Molecular Devices) and recorded using borosilicate glass micropipettes (World Precision Instruments) with DC resistance of 5–8 MΩ and PowerLab Hardware and LabChart 7 data acquisition software (ADInstruments). Spontaneous excitatory postsynaptic currents (sEPSCs) and spontaneous inhibitory postsynaptic currents (sIPSCs) were recorded in the voltage-clamp configuration at −60 and 0 mV, respectively, using a cesium methanesulfonate-based internal solution containing the following (in mM): 140 cesium methanesulfonate, 10 HEPES, 5 NaCl, 0.2 EGTA, 2 Mg-ATP, and 0.3 Na-GTP (280–290 mOsm), pH 7.25. Synaptic events over a 120 s epoch were analyzed using a custom MATLAB script. The frequency, amplitude, and decay of synaptic events were measured for each cell and averaged across experimental groups. Current-clamp recordings were performed in the *I* = 0 mode using a potassium gluconate-based internal solution containing the following (in mM): 130 K-gluconate, 10 KCl, 4 NaCl, 10 HEPES, 0.1 EGTA, 2 Mg-ATP, 0.3 Na-GTP (280–290 mOsm/L H_2_O), pH 7.25. Input–output curves were generated in response to a series of current injections from 0 to 150 pA in 10 pA steps. Rheobase was measured in response to a −70–0 mV current ramp. The resonant frequency profiles of principal neurons and PV interneurons were measured in response to a chirp stimulus. Subthreshold membrane properties and threshold firing of each neuron were measured in response to a sinusoidal chirp current of varying frequency from 0 to 60 Hz over a 60 s period for the subthreshold chirp and over a 3 or 20 s period for the suprathreshold chirp. The maximum amplitude response, peak power, and firing threshold over this frequency range were determined as a measure of the preferred resonant frequency of each neuron and averaged across neurons between experimental groups. Intrinsic electrophysiological properties, including input resistance, impedance, and whole cell time constant were measured for each neuron. Cells were eliminated from analysis if the series resistance was >20 MΩ or changed >20% over the course of the experiment.

### Stereotaxic surgery

Mice were anesthetized with a ketamine (90 mg/kg, i.p.)/xylazine (5–10 mg/kg, i.p.) cocktail and treated with sustained release buprenorphine (0.5–1.0 mg/kg, s.c.) prior to surgical procedures. To generate chronically epileptic mice, 100 nl of 20 mM kainic acid (Sigma-Aldrich, #K0250; dissolved in saline) was stereotaxically injected into the ventral hippocampus (AP −3.60 mm, ML −2.80 mm, DV −2.80 mm from the dura; Zeidler, et al., 2018). Control mice were injected with 100 nl of saline in the ventral hippocampus. For local field potential (LFP) recordings, mice were implanted with a custom head mount (Pinnacle Technology #8201) modified to include a depth electrode (PFA-coated stainless steel wire, A-M Systems) which was stereotaxically implanted into the BLA (AP −1.35 mm, ML 3.30 mm, DV −4.50 mm from the dura). Stainless steel screws served as a reference lead and an animal ground. EMG wires were also positioned in the neck muscles. Mice were allowed to recover for a minimum of 5 d prior to experimentation. For the PV ablation experiments, mice were stereotaxically injected with either a control virus rAAV2-mCherry, CMV-β-globin-DIO-mCherry-DTA-hGH pA (AAV-Flex-DTA; generated by Dr. Patrick M. Fuller, Harvard Medical School), or pAAV-mCherry-flex-dtA (Addgene, #58536) into the BLA using a Hamilton syringe and the stereotaxic coordinates above. Experiments were performed at 3 weeks following injection to allow for optimal virus expression and PV ablation.

### EEG/LFP recordings

Electroencephalogram (EEG) and LFP recordings were performed as previously described (Antonoudiou et al., 2022; DiLeo et al., 2022). EEG and LFP recordings in the frontal cortex and BLA, respectively, were recorded at 4 KHz and amplified 100× and acquired using the LabChart software (ADInstruments). For the analysis, the raw data were bandpass filtered (1–300 Hz), and spectral analysis was performed using custom analysis script developed in MATLAB using MatWAND (https://github.com/pantelisantonoudiou/MatWAND; Antonoudiou et al., 2022). Briefly, the recordings were divided into 5 s overlapping segments, and the power spectral density for a range of frequencies was obtained (Oppenheim et al., 1999) utilizing a fast Fourier transform similar to previous reports (Kruse and Eckhorn, 1996; Frigo and Johnson, 1998; Pape et al., 1998; Freeman et al., 2000).

For a subset of surgically implanted LFP mice, a brief restraint stress consisting of immobilization in a 50 ml falcon tube with a 0.25 in breathing hole drilled into the end for 30 min was performed. LFP recordings were conducted for 1 h prior to the restraint stress, and mice were returned to their recording chambers following the restraint stress where poststress LFP recordings were conducted for an additional 2 h. These poststress LFP were split into 30 min segments and normalized to the 1 h baseline to examine any stress-induced changes in local network function.

### Statistical analysis

Data were analyzed using the Prism 8/10 software (GraphPad) and custom scripts developed in MATLAB (MathWorks) or Python. Statistical significance between two experimental groups was determined using a Student’s *t* test. For the power analysis across specific frequency bands, a post hoc Šídák’s multiple-comparison test was performed to determine statistical significance. *p* < 0.05 were considered statistically significant, with 1–4 symbols used in figures to denote a significance level of *p* < 0.05, *p* < 0.01, *p* < 0.001, and *p* < 0.0001, respectively.

## Results

### Behavioral comorbidities in chronically epileptic mice

**Figure 1.**
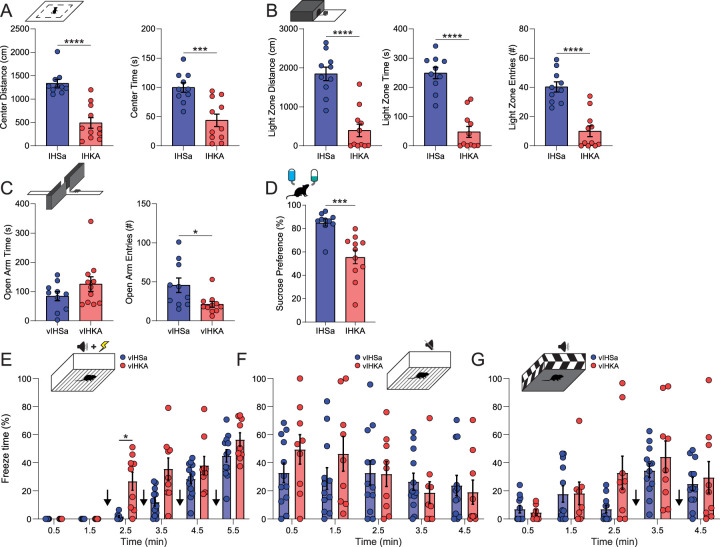
Anxiety-like behaviors in chronically epileptic mice. ***A***, Schematic of the open-field test (top) in which IHKA mice traveled less distance (left) and spent less time (right) in the center than IHSa mice. ***B***, Schematic of the light/dark box (top) in which IHKA mice traveled within (left), stayed (middle), and entered (right) significantly less time in the light zone than IHSa. ***C***, Schematic of the elevated plus maze (top) in which IHSa mice spent the same amount of time in the open arm as IHKA mice (left) but entered the open arm to explore more frequently (right). ***D***, Schematic of the sucrose preference test (top) showing IHKA mice lack hedonic sucrose-seeking behavior. ***E***, Schematic of the fear conditioning paradigm during which tone–shock pairing was conducted to induce a fear memory, and the representation of that memory was assayed in contextual (***F***) and cued (***G***) recall paradigms on a later day. * denotes the degree of significance. ***A–D***, Significance from unpaired two-tailed *t* test, ***E–G*** Significance from two-way ANOVA with Šídák’s multiple-comparison test. Data shown as mean ± SEM.

Chronically epileptic mice were generated following a single 100 nl injection of 20 mM kainic acid unilaterally into the hippocampus of male C57Bl/6J mice. All mice in the IHKA group were chronically epileptic with verified SRS. Seizure frequency was quantified in a subset of mice which exhibited an average of 1.39 ± 0.20 seizures per day compared with 0.00 ± 0.00 in IHSa controls. Avoidance behaviors were assessed in chronically epileptic mice 60 d postkainic acid injection using the open-field test, light/dark box, and elevated plus maze. Anhedonia was also assessed using the sucrose preference test and fear learning was measured using fear conditioning. Chronically epileptic male mice exhibit a decrease in both the amount of time spent (IHKA, 43.7 ± 10.6 s; IHSa, 99.8 ± 8.2 s; *p* = 0.0006; unpaired two-tailed *t* test) and distance traveled (IHKA, 489.5 ± 112.7 cm; IHSa, 1,336.3 ± 83.0 cm; *p* < 0.0001; unpaired two-tailed *t* test) while exploring the center of the open field compared with saline-injected controls (Fig. 1*A*; *N* = 10–11 mice per experimental group). Similarly, chronically epileptic male displayed an aversion to exploring the light chamber of the light/dark box, covering less ground (IHSa, 1,846.6 ± 173.4 cm; IHKA, 396.9 ± 160.6 cm; *p* < 0.0001; unpaired two-tailed *t* test), spending less time within (IHSa, 249.0 ± 19.5 s; IHKA, 47.8 ± 18.6 s; *p* < 0.0001; unpaired two-tailed *t* test), and making fewer entries into the light chamber (IHSa, 40.3 ± 3.5; IHKA, 10.0 ± 3.7; *p* < 0.0001; unpaired two-tailed *t* test) than controls (Fig. 1*B*, left, middle, right; *N* = 10–11 mice per experimental group). In the elevated plus maze, chronically epileptic male mice make a decreased number of entries into the open arm compared with controls (IHSa, 45.6 ± 9.3 entries; IHKA, 21.0 ± 3.7 entries; *p* = 0.019; unpaired two-tailed *t* test), without a significant difference in the amount of time spent in the open arm (IHSa, 84.2 ± 15.4 s; IHKA, 125.3 ± 25.0 s; *p* = 0.19; unpaired two-tailed *t* test; Fig. 1*C*; *N* = 10–11 mice per experimental group). Collectively, these data demonstrate an increase in avoidance behaviors in chronically epileptic male mice compared with controls. Chronically epileptic mice also exhibit increased anhedonia, exhibited by a decrease in sucrose preference compared with controls (Fig. 1*D*; IHSa, 85.14 ± 3.03%; IHKA, 55.73 ± 5.81%; *p* = 0.0003; unpaired two-tailed *t* test; *N* = 10–11 mice per experimental group), with chronically epileptic mice demonstrating no preference relative to chance (50%). The behavioral expression of fear is also altered in chronically epileptic mice. IHKA mice exhibit an increase in freezing during fear conditioning training (0.5, 0.0 ± 0.0%; 1.5, 0.0 ± 0.0% %; 2.5, 26.9 ± 6.4%; 3.5, 35.7 ± 7.8%; 4.5, 38.1 ± 6.4%; 5.5, 56.5 ± 4.8%) compared with IHSa controls (0.5, 0.0 ± 0.0%; 1.5, 0.0 ± 0.0%; 2.5, 1.7 ± 0.7%; 3.5, 12.0 ± 2.8%; 4.5, 28.5 ± 3.4%; 5.5, 45.0 ± 4.4%; Fig. 1*E*; treatment, *F*_(1,19)_ = 11.11; *p* = 0.0035; mixed-effect analysis; *N* = 8–12 mice per experimental group). IHKA mice however exhibit a similar degree of freezing during contextual fear recall (0.5, 49.6 ± 10.5%; 1.5, 46.4 ± 12.3%; 2.5, 32.1 ± 8.9%; 3.5, 18.8 ± 7.7%; 4.5, 19.1 ± 8.6%) and cued fear recall (0.5, 4.6 ± 2.0%; 1.5, 18.3 ± 8.1%; 2.5, 32.8 ± 11.8%; 3.5, 44.3 ± 11.3%; 4.5, 29.7 ± 11.2%) compared with IHSa controls (contextual: 0.5, 32.8 ± 7.0%; 1.5, 28.8 ± 7.7%; 2.5, 32.8 ± 8.6%3.5, 26.5 ± 6.1%; 4.5, 23.9 ± 7.2%; cued: 0.5, 7.0 ± 2.7%; 1.5, 17.8 ± 5.8%; 2.5, 7.1 ± 2.7%; 3.5, 34.5 ± 5.0%; 4.5, 25.0 ± 4.7%; Fig. 1*F*; treatment, *F*_(1,19)_ = 0.15; *p* = 0.70; mixed-effect analysis; Fig. 1*G*; treatment, *F*_(1,19)_ _=_ 1.44; *p* = 0.25; mixed-effect analysis; *N* = 7–12 mice per experimental group).

### PV interneuron deficits in the BLA of chronically epileptic mice

**Figure 2.**
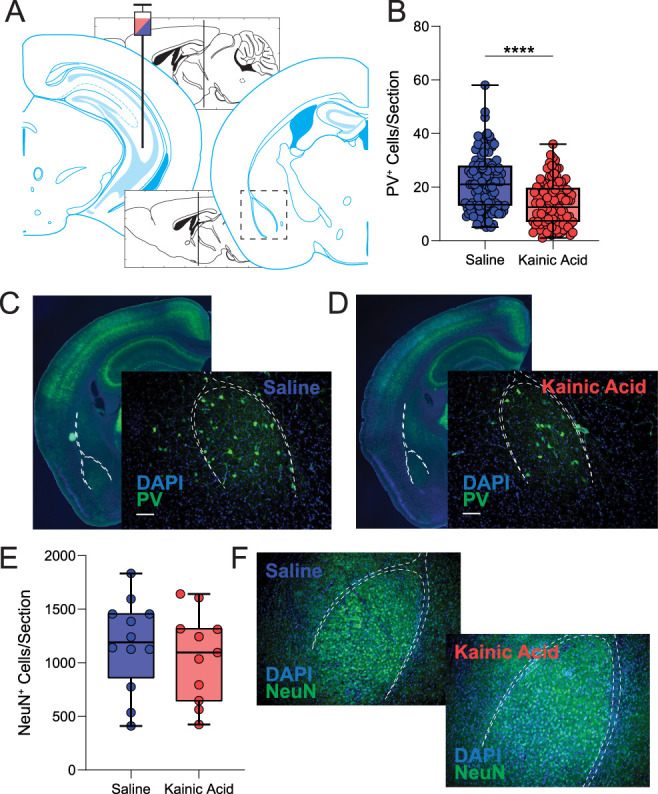
Loss of PV + BLA interneurons following IHKA treatment. ***A***, Schematic showing the intrahippocampal stereotaxic injection site and a slice through the BLA adapted from Franklin and Paxinos (2008). ***B***, Kainic acid significantly reduced the number of PV^+^ neurons counted within the BLA. ***C***, ***D***, Representative gross and close-up fluorescent IHC sections showing a decrease in the number of PV^+^ neurons in the BLA (white-dashed outline) of IHKA mice (***D***) compared with IHSa mice (***C***). NeuN staining (***E***) did not reveal a significant loss of mature neurons across the BLA of IHKA-treated mice compared with IHSa controls (***F***). * denotes the degree of significance between conditions measured by unpaired two-tailed *t* test. Data shown as a box-and-whisker plot with whiskers showing min and max.

To assess whether there is a loss of PV-positive interneurons in the BLA of chronically epileptic mice, we performed immunohistochemistry for PV in C57Bl/6J males 60 d following intrahippocampal injection of either saline or kainic acid (Fig. 2*A*). The average number of PV interneurons in the BLA is significantly reduced in chronically epileptic mice compared with controls (IHSa, 21.52 ± 0.95 cells/section; IHKA, 13.60 ± 0.73 cells/section; *p* < 0.0001; unpaired two-tailed *t* test; Fig. 2*B*; *n* = 120–122 sections, from *N* = 8 mice per experimental group), which can be appreciated in the representative images from saline- (Fig. 2*C*) and kainic acid-injected mice (Fig. 2*D*). However, we do not observe a significant decrease in NeuN expression across the BLA of chronically epileptic mice compared with controls (IHSa, 1,173 ± 122.3 cells/section; IHKA, 1,062 ± 124.2 cells/section; *p* = 0.53; unpaired two-tailed *t* test; Fig. 2*E*; *n* = 11–12 sections, from *N* = 2 mice per experimental group), which can be appreciated in the corresponding representative images (Fig. 2*F*).

**Figure 3.**
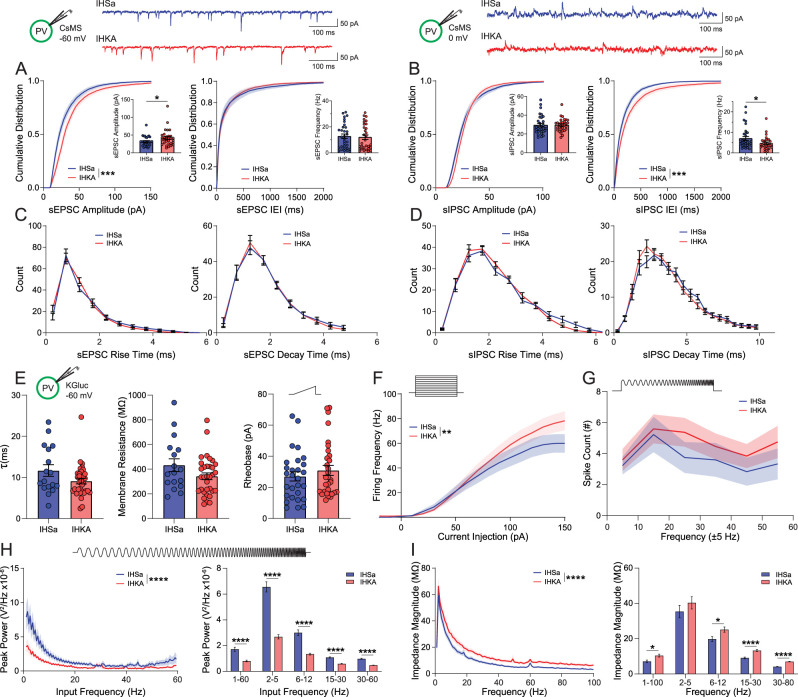
Active and passive electrophysiological properties of genetically identified PV + BLA interneurons. Schematics showing holding current, internal solution and PV-GFP+ neuron and representative IHSa and IHKA traces of afferent spontaneous excitatory (***A***) and inhibitory (***B***) postsynaptic currents from a whole-cell voltage–clamp recording (top). Excitatory afferent synaptic events (***A***) in IHKA exhibited a right-shift in the distribution of amplitudes (left) and an increase in the mean amplitude (inset), with no significant change to interevent intervals (IEI, right), mean frequency (inset), or the rise and decay kinetics (***C***). Inhibitory afferent synaptic events (***B***) in IHKA showed no change in the distribution of amplitudes (left) or mean amplitude (inset) but showed a right-shift in the distribution of IEI (right) and a decrease in the mean sIPSC frequency (inset), with no changes to rise or decay kinetics (***D***). ***E***, Intrinsic membrane properties including the membrane time constant (left), membrane resistance (middle), and rheobase (right) are not significantly altered in IHKA mice. ***F***, In response to successive depolarizing current steps (top), the firing frequency is increased in PV^+^ cells from IHKA mice at higher levels of current injection. ***G***, A chirp stimulation consisting of an accelerating sine wave on top of a depolarizing current step (top) did not reveal any significant differences between IHSa and IHKA in resonant firing output across the input frequencies tested. ***H***, A subthreshold chirp current (top) was injected into the current-clamped neuron to determine intrinsic passive resonant membrane properties at different input frequencies; IHKA neurons exhibited reduced resonant properties across the wavelengths tested (left) and within specific low-frequency bands (right). I, Membrane impedance as a function of frequency was significantly higher in IHKA (left) especially in higher frequency bands (right). * denotes the degree of significance between conditions. Cumulative distributions in ***A*** and ***B*** derive significance from Kolmogorov–Smirnov tests. The mean comparisons in ***A***, ***B***, and ***E*** derive significance from unpaired two-tailed *t* tests. Histograms in ***C*** and ***D*** derive significance from Wilcoxon matched pairs signed rank test, two-tailed. The two-factor comparisons in ***F–I*** derive significance from two-way ANOVA with Šídák’s multiple-comparison test. Data shown as mean ± SEM. The electrophysiological changes in BLA principal neurons in chronically epileptic mice are shown in Extended Data Figure 3-1.

The function of remaining PV interneurons in the BLA was assessed using whole-cell patch–clamp recording. Although there was no difference in the average frequency (Fig. 3*A*, inset; IHSa, 16.11 ± 2.42 Hz; IHKA, 15.13 ± 2.47 Hz; *p* = 0.7759; unpaired two-tailed *t* test) of sEPSCs on PV interneurons in the BLA, there was an increase in the average amplitude (Fig. 3*A*, insets; IHSa, −31.04 ± 2.39 pA; IHKA, −40.71 ± 4.15 pA; *p* = 0.0462; unpaired two-tailed *t* test; *n* = 31–32 cells; 11–13 mice per experimental group), in agreement with an increase in the cumulative distribution of the amplitude but not the interevent interval (IEI) of sEPSCs (Fig. 3*A*; sEPSC cumulative amplitude, *p* = 0.0001; sEPSC cumulative IEI, *p* = 0.8464; Kolmogorov–Smirnov test). In contrast, there is a significant decrease in the frequency of sIPSCs in PV interneurons in the BLA of chronically epileptic mice (Fig. 3*B* inset; IHSa, 8.00 ± 1.18 Hz; IHKA, 5.12 ± 0.67 Hz; *p* = 0.0395; unpaired two-tailed *t* test) with no change in amplitude (IHSa, 28.47 ± 1.77 pA; IHKA, 28.63 ± 1.23 pA; *p* = 0.9391; unpaired two-tailed *t* test; Fig. 3*B*, insets; *n* = 31–32 cells; 11–13 mice per experimental group). In line with this reduction in inhibitory signaling, the cumulative distribution demonstrates no change in the amplitude but a significant increase in the IEI of sIPSCs onto PV interneurons in the BLA of chronically epileptic mice (Fig. 3*B*; amplitude, *p* = 0.270; IEI, *p* = 0.0010; Kolmogorov–Smirnov test). However, there is no change in the distribution of the rise or decay times of either sEPSCs or sIPSCs between groups (Fig. 3*C*,*D*; sEPSC rise, *W* = −32; *p* = 0.2334; sEPSC decay, *W* = 1; *p* > 0.9999; sIPSC rise, *W* = −1; *p* = 0.9999; sIPSC decay, *W* = −20; *p* = 0.7285; Wilcoxon matched pairs signed rank test, two-tailed).

10.1523/ENEURO.0482-23.2024.f3-1 Figure 3-1 **Active and passive electrophysiological properties of BLA Principal neurons.** A. Whole-cell patch clamp recordings were taken from BLA principal neurons, and the cumulative distribution of sEPSCs in IHKA mice were observed to have a right-shift in amplitude and a left-shift in IEI, with a corresponding increase in mean amplitude, but no significant difference in mean frequency. B. BLA principal neurons showed a right-shift in the cumulative distribution of sIPSC amplitude in IHKA mice with no change in sIPSC IEI, with no significant changes in the mean amplitude or frequency of sIPSC afferents. Both sEPSCs and sIPSCs saw no significant changes in rise (C) or decay (D) kinetics between IHSa and IHKA mice. E, Intrinsic membrane properties including the membrane time constant (left), membrane resistance (middle), and rheobase (right) are not significantly altered in BLA principal neurons in IHKA mice. Firing properties including the input-output relationship (F) and firing in response to a supra-threshold chirp stimulation (G) did not reveal any significant differences between IHSa and IHKA mice. H, A subthreshold Chirp current (top) was injected into the current-clamped principal neuron to determine intrinsic passive resonant membrane properties at different input frequencies, IHKA principal neurons exhibited increased resonant properties across the wavelengths tested (left) and within specific high-frequency bands (right). I, Membrane impedance as a function of frequency was not significantly higher in IHKA across the entire frequency range (left) however there were some higher frequency bands that were significantly increased (right). * denotes the degree of significance between conditions. Cumulative distributions in A, B derive significance from Kolmogorov-Smirnov tests. The mean comparisons in A, B, and E derive significance from unpaired 2-tailed t-tests. Histograms in C, D derive significance from Wilcoxon matched pairs signed rank test, 2-tailed. The two-factor comparisons in F-I derive significance from 2-way ANOVA with Šídák's multiple comparisons test. Data shown as Mean ± SEM. Download Figure 3-1, TIF file.

To evaluate potential changes in the intrinsic properties of PV interneurons, we measured the membrane time constant (Fig. 3*E*, left; IHSa, 11.67 ± 1.41 ms; IHKA, 9.11 ± 0.69 ms; *p* = 0.0716; unpaired two-tailed *t* test), membrane resistance (Fig. 3*E*, middle; IHSa, 432.6 ± 51.4 MΩ; IHKA, 342.6 ± 27.4 MΩ; *p* = 0.0955; unpaired two-tailed *t* test), and rheobase (Fig. 3*E*, right; IHSa, 27.01 ± 2.94 pA; IHKA, 30.91 ± 3.21 pA; *p* = 0.377; unpaired two-tail *t* test). None of these measures were significantly altered in PV interneurons in BLA slices from control versus chronically epileptic mice (Fig. 3*E*; *n* = 17–33 cells; 5–10 mice per experimental group). Input–output curves in PV interneurons in the BLA demonstrate an increase in the frequency of spikes in response to higher current injections (Fig. 3*F*; treatment effects, *F*_(1,913)_ = 9.39; *p* = 0.0022; two-way ANOVA; *n* = 29–33 cells; 8–10 mice per experimental group) with a trend toward an increased number of spikes generated in response to a suprathreshold chirp stimulus (accelerating sine wave 0–60 Hz paired with a current step equal to rheobase to initiate firing) in chronically epileptic mice compared with controls (Fig. 3*G*; treatment effects, *F*_(1,276)_ = 2.88; *p* = 0.0906; two-way ANOVA; *n* = 18–30 cells; 510 mice per experimental group).

To assess potential changes in the resonant frequency profiles of PV interneurons to assess their ability to respond to oscillatory inputs, we measured the membrane response to a subthreshold chirp stimulus. PV interneurons in the BLA slices from chronically epileptic mice exhibit a decrease in peak power across frequencies (1–60 Hz, 8.05 × 10^−7^ ± 6.30 × 10^−8^ V^2^/Hz; 2–5 Hz, 2.68 × 10^−6^ ± 1.66 × 10^−7^ V^2^/Hz; 6–12 Hz, 1.34 × 10^−6^ ± 7.89 × 10^−8^ V^2^/Hz; 15–30 Hz, 5.90 × 10^−7^ ± 3.04 × 10^−8^ V^2^/Hz; 30–60 Hz, 4.74 × 10^−7^ ± 1.68 × 10^−8^ V^2^/Hz) compared with controls (1–60 Hz, 1.72 × 10^−6^ ± 1.55 × 10^−7^ V^2^/Hz; 2–5 Hz, 6.57 × 10^−6^ ± 3.99 × 10^−7^ V^2^/Hz; 6–12 Hz, 3.00 × 10^−6^ ± 2.21 × 10^−7^ V^2^/Hz; 15–30 Hz, 1.08 × 10^−6^ ± 4.22 × 10^−8^ V^2^/Hz; 30–60 Hz, 9.78 × 10^−7^ ± 4.07 × 10^−8^ V^2^/Hz; Fig. 3*H*; treatment effects, *F*_(1,6844)_ = 309.0; *p* < 0.0001; two-way ANOVA; *n* = 27–33 cells, 7–10 mice per experimental group). The impedance across frequencies is increased in PV interneurons in the BLA from chronically epileptic mice compared with controls (Fig. 3*I*; treatment effects, *F*_(1,5800)_ = 305.0; *p* < 0.0001; two-way ANOVA; *n* = 27–33 cells; 7–10 mice per experimental group) with statistical differences measured at 1–100 Hz (IHSa, 9.60 ± 1.00 MΩ; IHKA, 13.92 ± 1.10 MΩ), 6–12 Hz (IHSa, 26.40 ± 1.88 MΩ; IHKA, 33.56 ± 1.98 MΩ), 15–30 Hz (IHSa, 12.19 ± 0.66 MΩ; IHKA, 17.72 ± 0.75 MΩ), and 30–80 Hz (IHSa, 5.55 ± 0.18 MΩ; IHKA, 9.36 ± 0.21 MΩ; Fig. 3*I*; *n* = 27–33 cells, 7–10 mice per experimental group). These data suggest that the ability of PV interneurons to appropriately respond to oscillatory states or be recruited into generating oscillatory states may be deficient in chronically epileptic mice.

The functional consequences of the loss of and dysfunction of remaining PV interneurons in the BLA were assessed using whole-cell patch–clamp recording in principal neurons in the BLA. The cumulative distribution of the amplitude of sEPSCs is shifted toward the right, and the average amplitude of sEPSCs is increased in principal neurons in the BLA in slices from chronically epileptic mice compared with controls (Extended Data Fig. 3-1*A*; sEPSC amplitude distribution: *p* < 0.0001; Kolmogorov–Smirnov test; sEPSC mean amplitudes: IHSa, −19.85 ± 1.50 pA; IHKA, −25.78 ± 1.35 pA; *p* = 0.0073; unpaired two-tailed *t* test; *n* = 14–23 cells; 4–5 mice per experimental group) with no change in the distribution of the rise or decay times (Extended Data Fig. 3-1*C*; sEPSC rise, *W* = −18; *p* = 0.5186; sEPSC decay, *W* = 13, *p* = 0.5566; Wilcoxon matched pairs rank signed test, two-tailed; *n* = 14–23 cells; 4–5 mice per experimental group). Although there is no difference in the average frequency of sEPSCs in principal neurons in the BLA of chronically epileptic mice compared with controls, there is a leftward shift in the cumulative distribution of the IEI (Extended Data Fig. 3-1*A*; sEPSC IEI distribution: *p* = 0.0005; Kolmogorov–Smirnov test; sEPSC mean frequency: IHSa, 4.08 ± 0.92 Hz; IHKA, 6.56 ± 1.26 Hz; *p* = 0.1701; unpaired two-tailed *t* test; *n* = 14–23 cells; 4–5 mice per experimental group).

In contrast, while there is no significant difference in the average amplitude (IHSa, 27.50 ± 1.92 pA; IHKA, 33.62 ± 2.43 pA; *p* = 0.0780; unpaired two-tailed *t* test) or frequency (IHSa, 6.90 ± 1.40 Hz; IHKA, 6.96 ± 0.78 Hz; *p* = 0.9680; unpaired two-tailed *t* test) of sIPSCs (Extended Data Fig. 3-1*B*, insets; *n* = 14–21 cells; 4–5 mice per experimental group), there is a rightward shift in the cumulative distribution of the peak amplitude of sIPSCs and no change in the IEI in principal BLA neurons in slices from chronically epileptic mice compared with controls (Extended Data Fig. 3-1*B*; sIPSC cumulative amplitude, *p* < 0.0001; sIPSC cumulative IEI, *p* = 0.8643; Kolmogorov–Smirnov test) with no change in the distribution of rise or decay times (Extended Data Fig. 3-1*D*; sIPSC rise, *W* = −16; *p* = 0.5693; sIPSC decay, *W* = 13; *p* = 0.8194; Wilcoxon matched pairs rank signed test, two-tailed; *n* = 14–21 cells; 4–5 mice per experimental group).

The intrinsic properties of BLA principal neurons are not altered between control and chronically epileptic mice (Extended Data Fig. 3-1*E*–*I*). There is no difference in the membrane time constant (IHSa, 13.12 ± 1.15 ms; IHKA, 14.77 ± 1.66 ms; *p* = 0.4330; unpaired two-tailed *t* test), membrane resistance (IHSa, 291.3 ± 35.7 MΩ; IHKA: 390.5 ± 43.8 MΩ; *p* = 0.0942; unpaired two-tailed *t* test), or rheobase (IHSa, 40.84 ± 4.49 pA; IHKA, 32.20 ± 3.44 pA; *p* = 0.1390; unpaired two-tailed *t* test) between control and chronically epileptic mice (Extended Data Fig. 3-1*E*; *n* = 17–21 cells; 5–7 mice per experimental group). There was also no difference in the input–output curves (Extended Data Fig. 3-1*F*; treatment effects, *F*_(1,612)_ = 1.90; *p* = 0.169; two-way ANOVA) or number of spikes generated in response to the chirp stimulus (Extended Data Fig. 3-1*G*; treatment effects, *F*_(1,210)_ = 0.956; *p* = 0.3290; two-way ANOVA; *n* = 18–19 cells; 5–7 mice per experimental group). However, BLA principal neurons from chronically epileptic mice exhibited enhanced responsivity to the chirp stimulus (1–60 Hz, 6.63 × 10^−7^ ± 4.24 × 10^−8^ V^2^/Hz; 2–5 Hz, 1.90 × 10^−6^ ± 9.35 × 10^−8^ V^2^/Hz; 6–12 Hz, 1.08 × 10^−6^ ± 5.39 × 10^−8^ V^2^/Hz; 15–30 Hz, 5.45 × 10^−7^ ± 1.49 × 10^−8^ V^2^/Hz; 30–60 Hz, 4.17 × 10^−7^ ± 9.05 × 10^−9^ V^2^/Hz) compared with controls (1–60 Hz, 4.31 × 10^−7^ ± 4.24 × 10^−8^ V^2^/Hz; 2–5 Hz, 1.72 × 10^−6^ ± 1.27 × 10^−7^ V^2^/Hz; 6–12 Hz, 8.04 × 10^−7^ ± 4.90 × 10^−8^ V^2^/Hz; 15–30 Hz, 3.16 × 10^−7^ ± 1.38 × 10^−8^ V^2^/Hz; 30–60 Hz, 1.88 × 10^−7^ ± 2.92 × 10^−9^ V^2^/Hz; Extended Data Fig. 3-1*H*; treatment effects, *F*_(1,4248)_ = 117.6; *p* < 0.0001; two-way ANOVA; *n* = 18–20 cells; 5–7 mice per experimental group) with an increase in impedance observed at 15–30 Hz (IHSa, 7.16 ± 0.45 MΩ; IHKA, 9.13 ± 0.54 MΩ) and 30–80 Hz (IHSa, 3.20 ± 0.11 MΩ; IHKA, 3.92 ± 0.15 MΩ; Extended Data Fig. 3-1*I*; treatment effects, *F*_(1,3600)_ = 32.43; *p* < 0.0001; two-way ANOVA; *n* = 18–20 cells; 5–7 mice per experimental group). These data suggest that BLA principal neurons from chronically epileptic mice receive altered synaptic inputs and inappropriately respond to oscillatory inputs.

### Altered BLA network activity in chronically epileptic mice

**Figure 4.**
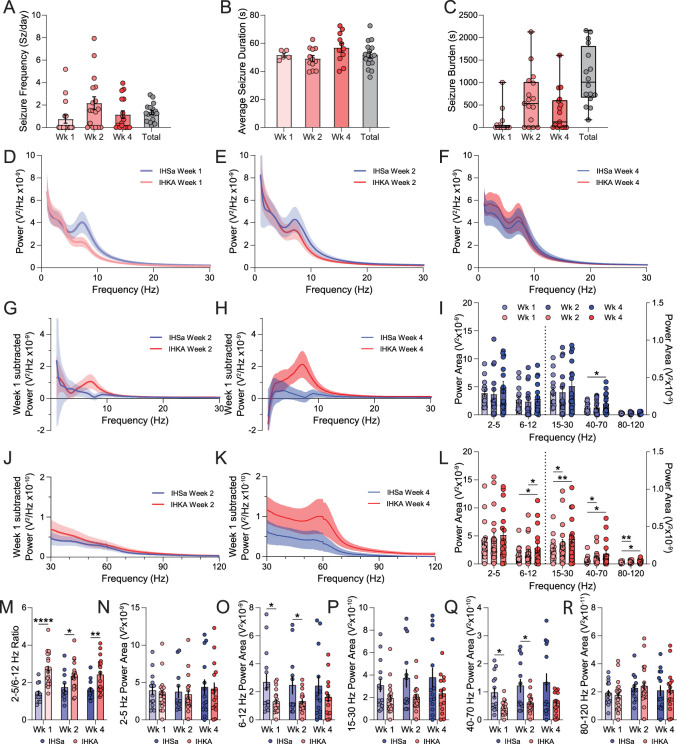
Intra-BLA LFP depth electrode recordings during development of TLE. From a depth electrode placed within the BLA in mice injected with IHKA, measures including seizure frequency (***A***), average seizure duration (***B***), and seizure burden (***C***) were analyzed over a period of 4 weeks. During interictal periods in IHKA mice, the LFP power spectra were analyzed across those same timepoints and compared with the power spectra of IHSa mice to examine the effects of epilepsy progression on basal power spectral properties in IHKA mice (***D–F***). To better visualize these changes, the power spectra were split into lower (***G***, ***H***) and higher (***J***, ***K***) frequency ranges from Wks 2 and 4 and were normalized to their Wk 1 power. The power area was binned into frequency bands in IHSa (***I***) and IHKA (***L***) mice, and frequency bands higher than 15 Hz were visualized on the right axis. IHKA mice exhibited significant progression across weeks within frequency bands during the development of TLE. To compare changes to power area across weeks resulting from IHKA treatment compared with IHSa, direct comparisons for each week between conditions were made within frequency bands with ratiometric comparisons (***M***) or raw power within bands (***N–R***) with notable significant differences observed in 2–5/6–12 Hz ratio, 6–12–30 Hz, and 40–70 Hz bands. * denotes the degree of significance between conditions. ***I***, ***L***, ***M–Q***, Significance from mixed-effect analysis with Šídák’s multiple-comparison test, data underwent outlier removal using the ROUT method (*Q* = 1%). Data shown as mean ± SEM except in ***C*** where data are displayed as box-and-whisker plot with whiskers showing min and max to emphasize all IHKA mice had seizure burden.

To examine whether there are changes in BLA network states in chronically epileptic mice, EEG and LFP recordings were performed in the cortex and BLA, respectively, for 4 weeks following either IHSa or IHKA injection. The average number of seizures per day [Fig. 4*A*; Week 1 (Wk 1), 0.79 ± 0.38; Wk 2, 2.30 ± 0.59; Wk 4, 1.22 ± 0.36; total, 1.39 ± 0.20], seizure duration (Fig. 4*B*; Wk 1, 51.61 ± 1.45 s; Wk 2, 49.11 ± 2.17 s; Wk 4, 56.91 ± 3.23 s; total, 52.60 ± 2.08 s), and total seizure burden (Fig. 4*C*; Wk 1, 105.94 ± 65.95 s; Wk 2, 653.12 ± 151.04 s; Wk 4, 381.56 ± 114.90 s; total, 1,140.63 ± 161.11 s) was measured at 1, 2, and 4 weeks post-IHKA injection (*N* = 16 mice). The power of the local field potential across frequencies was compared 1 week (Fig. 4*D*), 2 weeks (Fig. 4*E*), and 4 weeks (Fig. 4*F*) post-IHSa or IHKA injection (*N* = 14–18 mice per experimental group). The changes in the LFP with epilepsy progression can be appreciated from the power of the LFP subtracted from the LFP at Wk 1 (Fig. 4*G*,*H*,*J*,*K*). There are minimal changes in the LFP in the BLA over time in the IHSa group, with only modest increases in the 40–70 Hz range (Wk 1, 9.71 ± 1.76 × 10^−11^ V^2^; Wk 2, 9.94 ± 2.31 × 10^−11^ V^2^; Wk 4, 1.52 ± 0.33 × 10^−10^ V^2^; Fig. 4*I*; week effect, *F*_(1.38,94.89)_ = 3.58; *p* = 0.048; mixed-effect analysis; *N* = 12–14 mice). More profound changes in the LFP in the BLA were observed over time with epilepsy progression in IHKA group, with significance in the 6–12 Hz (Wk 1, 1.54 ± 0.29 × 10^−9^ V^2^; Wk 2, 2.16 ± 0.50 × 10^−9^ V^2^; Wk 4, 2.87 ± 0.75 × 10^−9^ V^2^), 15–30 Hz (Wk 1, 1.94 ± 0.31 × 10^−10^ V^2^; Wk 2, 2.75 ± 0.55 × 10^−10^ V^2^; Wk 4, 3.36 ± 0.67 × 10^−10^ V^2^), 40–70 Hz range (Wk 1, 5.23 ± 1.03 × 10^−11^ V^2^; Wk 2, 8.80 ± 1.98 × 10^−11^ V^2^; Wk 4, 1.39 ± 0.40 × 10^−10^ V^2^), and 80–120 Hz (Wk 1, 1.76 ± 0.24 × 10^−11^ V^2^; Wk 2, 2.41 ± 0.31 × 10^−11^ V^2^; Wk 4, 2.81 ± 0.58 × 10^−11^ V^2^; Fig. 4*L*; week effect, *F*_(1.48,133.6)_ = 8.53; *p* = 0.0012; mixed-effect analysis; *N* = 16–20 mice). The ratiometric comparison of power in the 2–5 and 6–12 Hz bands, a marker of BLA network state associated with fear and anxiety, was significantly elevated IHKA (Wk 1, 2.70 ± 0.19; Wk 2, 2.35 ± 0.17; Wk 4, 2.42 ± 0.21) compared with IHSa (Wk 1, 1.43 ± 0.14; Wk 2, 1.73 ± 0.22; Wk 4, 1.60 ± 0.14; Fig. 4*M*; week × treatment interaction, *F*_(2,58)_ = 3.80; *p* = 0.028; mixed-effect analysis; *N* = 12–20 mice). A direct comparison to changes in individual power bands between IHSa and IHKA mice (Fig. 4*N*–*R*) revealed changes in epilepsy progression in IHKA mice. There were no significant differences in the 2–5 Hz power band across weeks in both IHSa (Wk 1, 3.93 ± 0.63 × 10^−9^ V^2^; Wk 2, 3.75 ± 0.82 × 10^−9^ V^2^; Wk 4, 4.37 ± 1.05 × 10^−9^ V^2^) and IHKA mice (Wk 1, 3.47 ± 0.53 × 10^−9^ V^2^; Wk 2, 3.40 ± 0.59 × 10^−9^ V^2^; Wk 4, 4.15 ± 0.86 × 10^−9^ V^2^; Fig. 4*N*; treatment effect, *F*_(1,31)_ = 0.085; *p* = 0.77; mixed-effect analysis; *N* = 12–19 mice). Importantly, the IHSa 6–12 Hz power area (Wk 1, 2.69 ± 0.60 × 10^−9^ V^2^; Wk 2, 2.47 ± 0.64 × 10^−9^ V^2^; Wk 4, 2.42 ± 0.69 × 10^−9^ V^2^) was significantly higher than IHKA (Wk 1, 1.31 ± 0.18 × 10^−9^ V^2^; Wk 2, 1.32 ± 0.18 × 10^−9^ V^2^; Wk 4, 1.62 ± 0.35 × 10^−9^ V^2^; Fig. 4*O*; treatment effect, *F*_(1,31)_ = 4.20; *p* = 0.049; mixed-effect analysis; *N* = 12–19 mice), thereby driving the changes observed in Figure 4*M*. Power was nonsignificantly diminished in the 15–30 Hz band in IHKA (Wk 1, 1.94 ± 0.31 × 10^−10^ V^2^; Wk 2, 2.04 ± 0.27 × 10^−10^ V^2^; Wk 4, 2.33 ± 0.43 × 10^−10^ V^2^) compared with IHSa mice (Wk 1, 3.06 ± 0.58 × 10^−10^ V^2^; Wk 2, 3.71 ± 0.81 × 10^−10^ V^2^; Wk 4, 3.77 ± 0.88 × 10^−10^ V^2^; Fig. 4*P*; treatment effect, *F*_(1,32)_ = 2.53; *p* = 0.12; mixed-effect analysis; *N* = 13–20 mice). The low-gamma band from 40–70 Hz band in IHKA mice (Wk 1, 4.35 ± 0.56 × 10^−11^ V^2^; Wk 2, 6.13 ± 0.78 × 10^−11^ V^2^; Wk 4, 6.17 ± 1.05 × 10^−11^ V^2^) was significantly lower compared with IHSa (Wk 1, 9.71 ± 1.76 × 10^−11^ V^2^; Wk 2, 1.22 ± 0.25 × 10^−10^ V^2^; Wk 4, 1.34 ± 0.33 × 10^−10^ V^2^; Fig. 4*Q*; treatment effect, *F*_(1,31)_ = 7.40; *p* = 0.011; mixed-effect analysis; *N* = 12–20 mice). Power in the 80–120 high gamma band however was not different between IHSa (Wk 1, 1.91 ± 0.23 × 10^−11^ V^2^; Wk 2, 2.26 ± 0.27 × 10^−11^ V^2^; Wk 4, 2.09 ± 0.38 × 10^−11^ V^2^) and IHKA (Wk 1, 1.76 ± 0.24 × 10^−11^ V^2^; Wk 2, 2.41 ± 0.31 × 10^−11^ V^2^; Wk 4, 2.12 ± 0.37 × 10^−11^ V^2^; Fig. 4*R*; treatment effect, *F*_(1,32)_ = 0.023; *p* = 0.88; mixed-effect analysis; *N* = 12–20 mice). These data suggest BLA network dysfunction associated with epilepsy progression, which likely involves the loss of PV-positive interneurons in the BLA, which have been demonstrated to play a critical role in orchestrating oscillations in the BLA.

### Deficits in PV interneurons are sufficient to alter BLA network and behavioral states

**Figure 5.**
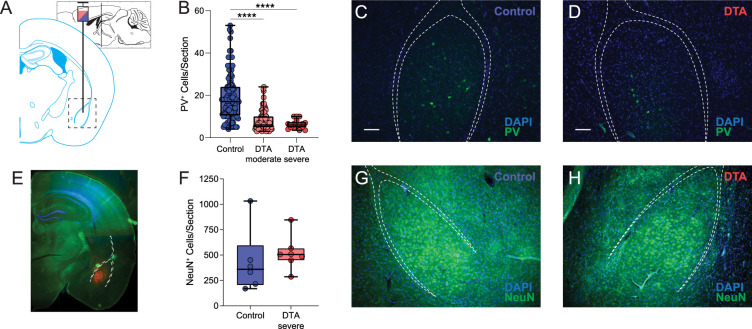
Genetically targeted viral ablation of PV^+^ interneurons in the BLA. ***A***, Schematic showing the stereotaxic injection site of an AAV2-mCherry (control) or AAV-Flex-DTA virus into the BLA adapted from Franklin and Paxinos (2008). ***B***, DTA specifically ablates PV^+^ interneurons within the BLA to recapitulate the PV^+^ cell loss observed in the vIHKA model. ***C***, ***D***, Representative fluorescent IHC sections showing a decrease in the number of PV^+^ neurons in the BLA (white-dashed outline) of DTA-injected mice compared with control virus-injected mice. The PV interneuron number was compared between mice with a modest versus severe behavioral phenotype. ***E***, Representative section showing mCherry fluorescence constrained within targeted BLA region. A summary graph showing no off-target effects caused by inappropriate action of the AAV-Flex-DTA virus on Cre-negative mature neurons. ***G***, ***H***, Representative NeuN-stained sections from the anterior BLA used for cell counting. * denotes the degree of significance between conditions measured by unpaired two-tailed *t* test. Data shown as a box-and-whisker plot with whiskers showing min and max.

To assess whether the loss of PV-positive interneurons in the BLA contributes to the deficits in network and behavioral states, we injected PV-Cre mice with either AAV-mCherry (control) or AAV-Flex-DTA (Fig. 5*A*) to partially ablate PV interneurons in the BLA and examined the impact network activity and behavioral states. There is a reduction in the number of PV-positive interneurons in the BLA of AAV-DTA mice compared with controls. Furthermore, we examined the loss of PV interneurons in the BLA in a subset of mice with a more severe phenotype (control, 18.90 ± 1.07 cells/section; DTA moderate, 7.96 ± 0.42 cells/section; DTA severe, 6.29 ± 0.52 cells/section; Fig. 5*B*; *n* = 17–111 sections; *N* = 5–6 mice per experimental group; *F*_(2,234)_ = 54.80; *p* < 0.0001; one-way ANOVA), which can be appreciated in the representative images from control (Fig. 5*C*) and AAV-DTA–injected mice (Fig. 5*D*). This manipulation is spatially restricted to the BLA (Fig. 5*E*) and appears to be specific for PV interneuron ablation since we do not observe any change in the number of SST interneurons (controls, 12.6 ± 0.7 cells/section; AAV-DTA, 13.0 ± 0.8 cells/section; *n* = 10 sections; *N* = 3–5 mice per experimental group; *p* = 0.75; unpaired two-tailed *t* test; not shown) or NeuN-positive neurons in the anterior portion of the BLA (controls, 431.5 ± 127.8 cells/section; AAV-DTA, 521.3 ± 63.6 cells/section) in the AAV-DTA group compared with controls (Fig. 5*F*–*H*; *n* = 6 sections; from *N* = 2–3 mice per experimental group; *p* = 0.52; unpaired two-tailed *t* test). Furthermore, we do not observe any difference in the number of PV interneurons in the thalamic reticular nucleus, a region which is enriched in PV interneurons and lies medial to the BLA (control, 243.0 ± 12.7; DTA, 231.3 ± 11.5), nor in the hippocampus, a region implicated in epilepsy (control, 25.3 ± 2.3; DTA, 25.6 ± 1.2; data not shown; *n* = 4–5 sections; from *N* = 2–3 mice per experimental group; *p* > 0.05).

**Figure 6.**
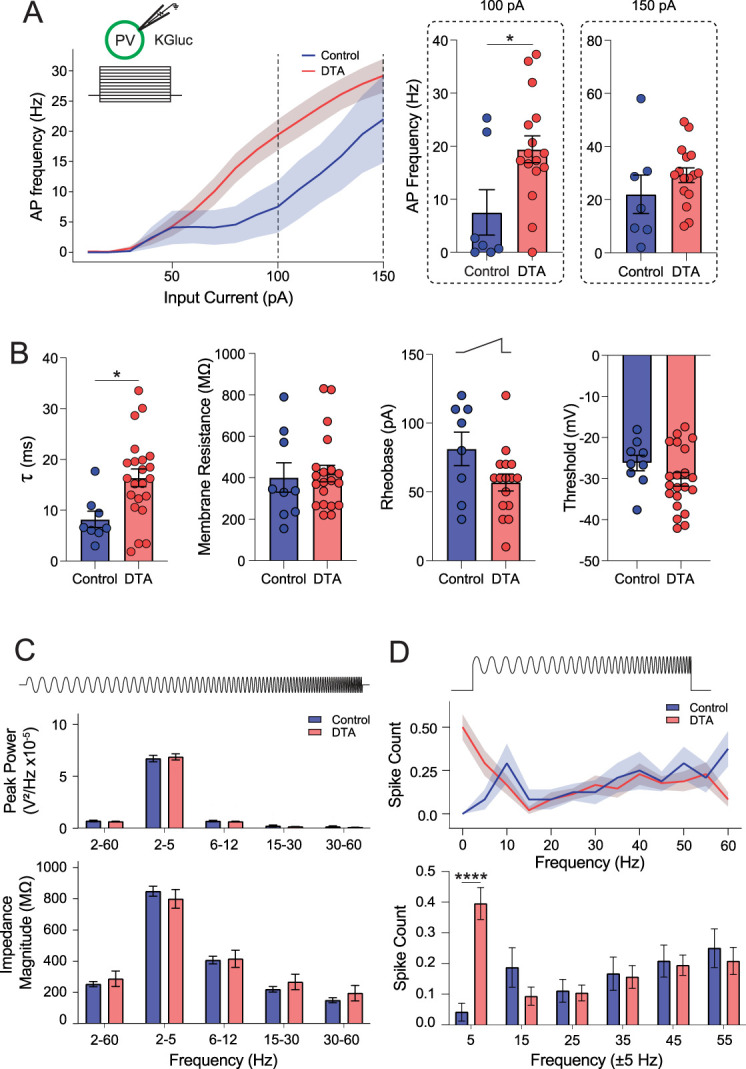
Partial loss of BLA PV^+^ interneurons shifts excitability and resonance. ***A***, Schematic showing internal solution and genetically identified PV^+^ interneurons. DTA data represent remaining PV^+^ interneurons postablation. Input–output curves demonstrate that partial ablation of PV^+^ interneurons drives increased output in response to a 100 pA, but not a 150 pA, depolarizing square wave. ***B***, The membrane time constant significantly increased in DTA mice (left); however other intrinsic membrane properties such as resistance (mid left), rheobase (mid right), and threshold (right) remain unaffected. ***C***, There were no differences in peak power (top) or membrane impedance (bottom) between control and DTA mice. ***D***, In response to a chirp stimulation consisting of an accelerating sine wave on top of a suprathreshold depolarizing current step, DTA mice demonstrated a significant treatment × frequency interaction with regard to spike generation. * denotes the degree of significance between conditions. The mean comparisons in ***A*** and ***B*** derive significance from unpaired two-tailed *t* tests. The two-factor comparisons in ***D*** derive significance from two-way ANOVA with Šídák’s multiple-comparison test. Data shown as mean ± SEM.

To examine whether the ablation of a large percentage of PV interneurons in the BLA impacts the function of remaining PV interneurons, we performed whole-cell patch–clamp recording and assessed the excitability and intrinsic membrane properties of the unablated PV interneurons. The input–output relationship in PV interneurons in the BLA is shifted in slices from AAV-DTA mice compared with controls (Fig. 6*A*). There is a significant increase in the number of action potentials fired in response to a 100 pA step in PV interneurons from AAV-DTA mice (19.4 ± 2.5 Hz) compared with controls (7.5 ± 4.3 Hz; *p* = 0.020; unpaired two-tailed *t* test), but not at 150 pA (Fig. 6*A*; control, 22.0 ± 7.2 Hz; DTA, 29.21 ± 2.77; *p* = 0.26; unpaired two-tailed *t* test; *n* = 7–16 cells; 3–5 mice per experimental group). The intrinsic membrane properties of PV interneurons in the BLA were compared between AAV-DTA mice and controls. There was a significant increase in the membrane time constant (control, 8.2 ± 1.6 ms; DTA, 16.4 ± 1.9 ms; *p* = 0.012; unpaired two-tailed *t* test) with no significant difference in membrane resistance (control, 401.4 ± 71.0 MΩ; DTA, 432.8 ± 44.0 MΩ; *p* = 0.81; unpaired two-tailed *t* test), rheobase (control, 81.3 ± 12.2 pA; DTA, 56.4 ± 7.2 pA; *p* = 0.059; unpaired two-tailed *t* test), or threshold (control, −26.2 ± 1.9 mV; DTA, −30.4 ± 1.9 mV; *p* = 0.17; unpaired two-tailed *t* test) between control and AAV-DTA mice (Fig. 6*B*; *n* = 8–22 cells; 3–5 mice per experimental group).

The ablation of PV interneurons in the BLA did not alter the response of remaining PV interneurons to the subthreshold chirp stimulus. No differences were observed in the peak power in PV interneurons from AAV-DTA mice (2–60 Hz, 0.63 ± 0.02 × 10^−5^ V^2^/Hz; 2–5 Hz, 6.85 ± 0.29 × 10^−5^ V^2^/Hz; 6–12 Hz, 0.63 ± 0.03 × 10^−5^ V^2^/Hz; 15–30 Hz, 0.14 ± 0.02 × 10^−5^ V^2^/Hz; 30–60 Hz, 0.06 ± 0.02 × 10^−5^ V^2^/Hz) compared with controls (2–60 Hz, 0.68 ± 0.08 × 10^−5^ V^2^/Hz; 2–5 Hz, 6.70 ± 0.32 × 10^−5^ V^2^/Hz; 6–12 Hz, 0.67 ± 0.08 × 10^−5^ V^2^/Hz; 15–30 Hz, 0.21 ± 0.09 × 10^−5^ V^2^/Hz; 30–60 Hz, 0.13 ± 0.08 × 10^−5^ V^2^/Hz; Fig. 6*C*, top; treatment effects, *F*_(1,26)_ = 0.0107; *p* = 0.92; two-way ANOVA; *n* = 8–20 cells; 3–5 mice per experimental group). Similarly, impedance was unchanged following DTA treatment (2–60 Hz, 253.1 ± 16.6 MΩ; 2–5 Hz, 849.0 ± 32.1 MΩ; 6–12 Hz, 407.2 ± 24.7 MΩ; 15–30 Hz, 219.9 ± 18.2 MΩ; 30–60 Hz, 149.8 ± 16.0 MΩ) compared with controls (2–60 Hz, 287.5 ± 49.7 MΩ; 2–5 Hz, 799.3 ± 60.0 MΩ; 6–12 Hz, 415.4 ± 55.3 MΩ; 15–30 Hz, 267.5 ± 50.0 MΩ; 30–60 Hz, 195.0 ± 49.9 MΩ; Fig. 6*C*; treatment effects *F*_(1,26) _= 0.159; *p* = 0.694; *n* = 8–20 cells; 3–5 mice per experimental group). PV interneurons in the BLA of AAV-DTA mice exhibit an increase in the number of spikes elicited in response to the suprathreshold chirp stimulus at low input frequencies (DTA: 5 Hz, 0.40 ± 0.05; 15 Hz, 0.09 ± 0.03; 25 Hz, 0.10 ± 0.03; 35 Hz, 0.16 ± 0.04; 45 Hz, 0.19 ± 0.03; 55 Hz, 0.21 ± 0.04) compared with controls (5 Hz, 0.04 ± 0.03; 15 Hz, 0.19 ± 0.06; 25 Hz, 0.11 ± 0.04; 35 Hz, 0.17 ± 0.05; 45 Hz, 0.21 ± 0.05; 55 Hz, 0.25 ± 0.06; Fig. 6*D*; frequency × treatment interaction, *F*_(5,132) _= 6.141; *p* < 0.0001; two-way ANOVA; *n* = 8–16 cells; 3–5 mice per experimental group). These data suggest that the function of remaining PV interneurons and the ability to coordinate network states are impaired in mice with ablation of PV interneurons in the BLA.

**Figure 7.**
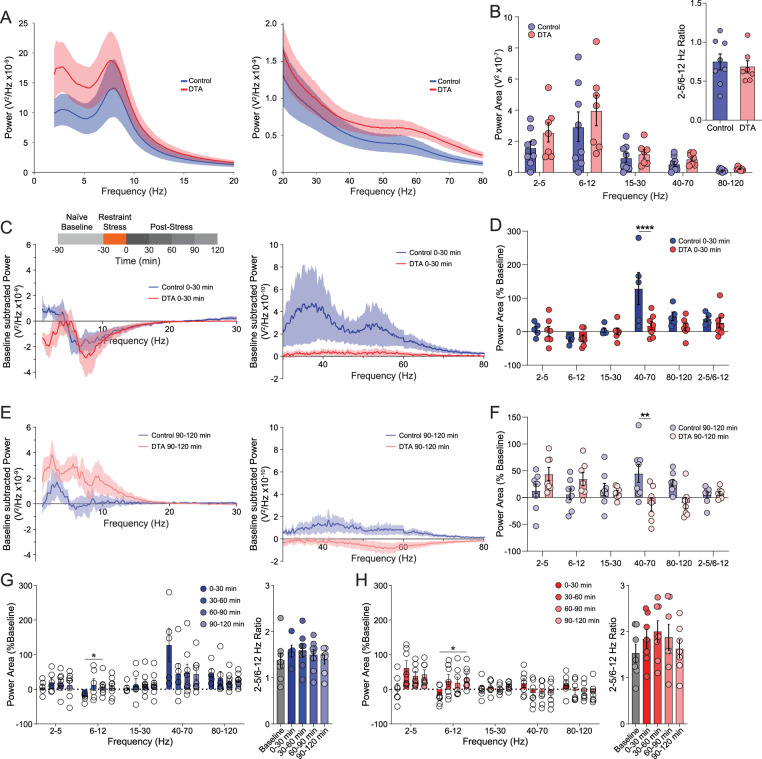
The loss of PV^+^ interneurons does not alter naive or stressed network states in control and AAV-DTA mice. ***A***, Power spectra from LFP recordings in the BLA of control and DTA-injected mice showing an elevated basal power spectrum following the loss of PV^+^ interneurons in the BLA. ***B***, However the increase in the power area in unstressed mice was not significantly altered by the local, specific loss of PV^+^ interneurons. Furthermore, the 2–5/6–12 Hz ratio (***B***, inset) remained unaltered between conditions at rest. To examine how a loss of PV^+^ interneurons in the BLA affected network oscillations following activation of the stress response, mice were exposed to an acute restraint stress (***C***, experimental schematic inset). The LFP was parsed into 30 min segments to examine the temporal component of stress-induced changes at 0–30 min (***C***, ***D***) and 90–120 min (***E***, ***F***) following the acute stress. Direct comparisons between control and AAV-DTA mice showing changes in low-gamma power at 0–30 min (***D***) that persisted to 90–120 min (***F***). Within treatment comparisons of different timepoints in control (***G***) and AAV-DTA (***H***) mice exhibiting no significant stress-induced changes in the power area compared with their baseline values. * denotes level of significance between labeled conditions and timepoints. ***D***, ***F***, Significance from Šídák’s multiple-comparison test. ***H***, Significance from two-way ANOVA with Šídák’s multiple-comparison test. ***G***, Significance from mixed-effect analysis with Šídák’s multiple-comparison test. Data shown as mean ± SEM.

The loss of PV interneurons in the BLA did not significantly alter the baseline BLA network activity (2–5 Hz, 2.57 × 10^−7^ ± 6.17 × 10^−8^ V^2^; 6–12 Hz, 3.98 × 10–7 ± 9.76 × 10–8 V^2^; 15–30 Hz, 1.19 × 10^−7^ ± 2.68 × 10^−8^ V^2^; 40–70 Hz, 8.61 × 10^−8^ ± 1.41 × 10^−8^ V^2^; 80–120 Hz, 2.22 × 10^−8^ ± 3.60 × 10^−9^ V^2^; 2–5/6–12 Hz, 0.68 ± 0.08) compared with controls (2–5 Hz, 1.59 × 10^−7^ ± 4.07 × 10^−8^ V^2^; 6–12 Hz, 2.93 × 10^−7^ ± 9.54 × 10^−8^ V^2^; 15–30 Hz, 9.47 × 10^−8^ ± 2.94 × 10^−8^ V^2^; 40–70 Hz, 5.43 × 10^−8^ ± 1.64 × 10^−8^ V^2^; 80–120 Hz, 1.31 × 10^−8^ ± 3.17 × 10^−9^ V^2^; 2–5/6–12 Hz, 0.75 ± 0.10; Fig. 7*A*,*B*; frequency × treatment interaction, *F*_(4,65)_ = 0.38; *p* = 0.820; two-way ANOVA; Fig. 7*B* inset, *p* = 0.638; unpaired two-tailed *t* test; *N* = 7–8 mice per experimental group). Although we did not observe any network changes under basal conditions, network states are altered in AAV-DTA mice in response to stress exposure, suggesting that the network is not engaged in the same manner under different contexts. A baseline, stress-naive LFP recording was taken in vivo prior to a 30 min restraint stress to compare against the poststress LFP recording at multiple poststress timepoints. Changes in the power area of pre- vs poststress timepoints were observed within control (enhanced 40–70 Hz band) and DTA (enhanced 2–5 Hz band) mice immediately (Fig. 7*C*) and up to 2 h following the stressor (Fig. 7*E*). Notably, a significant decrease was observed in the low-gamma range in the BLA of AAV-DTA–treated mice (40–70 Hz, 18.1 ± 12.9%) compared with control (40–70 Hz, 128.8 ± 48.1%) in the 30 min following the acute stress (Fig. 7*D*; *p* < 0.001, Holm–Šídák's multiple-comparison test; *N* = 5–8 mice), which remained diminished compared with control 90–120 min poststress (control 40–70 Hz, 45.1 ± 16.9%; DTA 40–70 Hz, −12.5 ± 12.5%; Fig. 7*F*; *p* = 0.002; Holm–Šídák's multiple-comparison test; *N* = 5–8 mice). There were no major changes in power compared with the prestress baseline within control (Fig. 7*G*, frequency × time interaction, *F*_(16,115)_ = 2.891; *p* = 0.0005; mixed-effect analysis; 7G inset, time effect, *F*_(1.464,9.152)_ = 2.859; *p* = 0.117; mixed-effect analysis; *N* = 5–8 mice) or AAV-DTA mice (Fig. 7*H*, frequency × time interaction, *F*_(16,120)_ = 5.793; *p* < 0.0001; two-way ANOVA; 7H inset, time effect, *F*_(1.539,9.233)_ = 3.608; *p* = 0.0775; one-way ANOVA; *N* = 7 mice). Repeated measures showed that in both control and DTA mice, there were significant changes in the 6–12 Hz band 0–30 min following the acute restraint stress which then rebounded and became elevated for the remainder of the recording, reaching significance at 60–90 min in control and 90–120 min in DTA mice (Fig. 7*G*,*H*).

**Figure 8.**
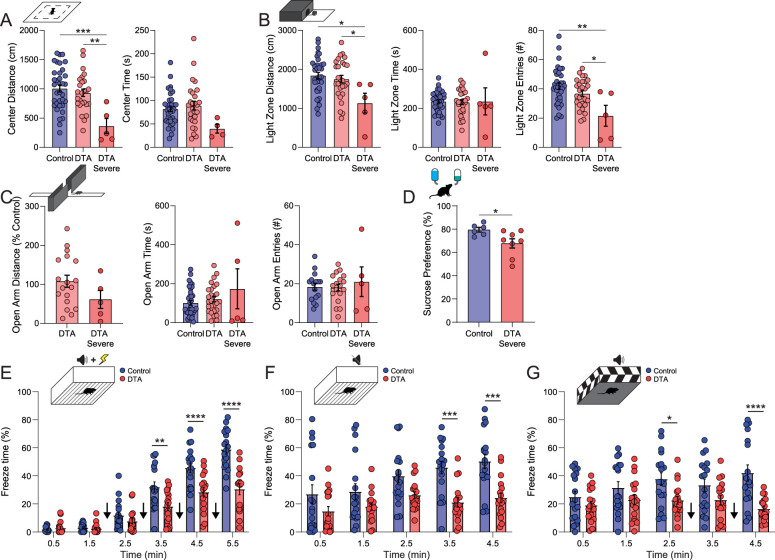
The specific loss of PV^+^ Interneurons impairs fear learning and recall of fear memories. ***A***, Schematic of the open-field test (top) in which both control and DTA mice explored the center of the open field significantly more than the DTA severe group. ***B***, Schematic of the light/dark box (top) in which both control and DTA mice entered and explored the light zone more than DTA severe mice. ***C***, Schematic of the elevated plus maze (top) in which DTA and DTA severe mice explored and spent time in the open arms to a similar degree. ***D***, Schematic of the sucrose preference test in which DTA severe mice exhibit anhedonia significantly. ***E***, Schematic of the fear conditioning paradigm (top) in which control and mice were subjected to four bouts of tone–shock pairing (black arrows), while the time spent immobile was recorded. Control mice froze significantly more after the first tone–shock pairing. ***F***, Schematic of the contextual recall paradigm (top) during which mice were placed into the fear conditioning chamber with the tone and shock disabled. Control mice froze longer than DTA mice across the time spent in the chamber, finally reaching significant levels in the final 2 min spent within the chamber. ***G***, Schematic of the cued recall paradigm (top) in which the tone associated with the shock is played (black arrows) while in an environment different from the fear conditioning paradigm. Control mice exhibited significantly more freezing behavior prior to the first tone presentation and continued to freeze significantly more upon tone presentation. * denotes degree of significance between conditions. ***A–D***, Significance from two-way ANOVA with Šídák’s multiple-comparison test. ***E–G***, Significance from mixed-effect analysis with Šídák’s multiple-comparison test, data underwent outlier removal using the ROUT method (*Q* = 1%). Data shown as mean ± SEM.

These data suggest that the ability to coordinate oscillations in the BLA may be altered in mice with a loss of PV interneurons in the BLA. Given that these oscillatory states govern behavioral states, we examine the impact on avoidance behaviors and the behavioral expression of fear (Fig. 8). Mice with a moderate AAV-DTA PV ablation show no difference in distance traveled or amount of time spent in the center of an open field compared with controls; however a subset of mice exhibited a more severe phenotype with a significant decrease in the distance traveled in the center of the open-field arena (control, 1,008.0 ± 61.9 cm; DTA, 932.5 ± 63.6 cm; DTA severe, 366.4 ± 127.2 cm; *F*_(2,63)_ = 7.55; *p* = 0.0012; one-way ANOVA) with no significant difference in the time spent exploring (control, 82.3 ± 5.9 s; DTA, 89.4 ± 9.3 s; DTA severe, 39.9 ± 9.4 s; *F*_(2,63)_ = 2.62; *p* = 0.081; one-way ANOVA; Fig. 8*A*; *N* = 4–27 mice per experimental group). Similarly, moderately affected AAV-DTA mice do not exhibit an aversion to exploring the light chamber of a light/dark box test compared with controls; however mice with a more severe DTA ablation exhibit a decrease in the degree of light chamber exploration (control, 1,843.0 ± 81.2 cm; DTA, 1,751.0 ± 97.5 cm; DTA severe, 1,135.0 ± 253.5 cm; *F*_(2,64)_ = 4.43; *p* = 0.016; one-way ANOVA), number of entries into the light chamber (control, 41.9 ± 2.2 entries; DTA, 36.9 ± 1.9 entries; DTA severe, 21.6 ± 7.2 entries; *F*_(2,63)_ = 6.61; *p* = 0.0025; one-way ANOVA), with no difference in the time spent in the light chamber (control, 235.9 ± 8.9 s; DTA, 231.9 ± 12.5 s; DTA severe, 235.8 ± 69.4 s; *F*_(2,64)_ = 0.027; *p* = 0.97; one-way ANOVA; Fig. 8*B*; *N* = 5–27 mice per experimental group). Comparing between moderate and severe DTA ablation, there is no significant difference in the distance traveled along the open arm of an elevated plus maze (DTA, 108.1 ± 15.7%; DTA severe, 61.5 ± 23.1%; *p* = 0.1641; unpaired two-tailed *t* test); similarly, there are no differences in either time spent exploring (control, 102.4 ± 12.1 s; DTA, 121.6 ± 14.3 s; DTA severe, 174.4 ± 102.4 s; *F*_(2,64)_ = 1.48; *p* = 0.24; one-way ANOVA) or the number of entries onto the open arm (control, 18.4 ± 1.8 entries; DTA, 18.0 ± 1.7 entries; DTA severe, 21.0 ± 7.6 entries; *F*_(2,36)_ = 0.22; *p* = 0.80; one-way ANOVA), compared with control (Fig. 8*C*; *N* = 5–27 mice per experimental group). Similar to what was observed in chronically epileptic mice, AAV-DTA mice exhibit anhedonic qualities when given access to sucrose water, showing weaker preference to sucrose than controls (control, 79.5 ± 2.0% preference; DTA, 67.8 ± 4.0% preference; *p* = 0.0357; unpaired two-tailed *t* test; Fig. 8*D*; *N* = 6–8 mice per experimental group). These data suggest that the degree of PV interneuron loss in the BLA may impact the expression of avoidance or reward-seeking behaviors. Furthermore, the loss of PV interneurons in the BLA does alter the behavioral expression of fear. AAV-DTA mice exhibit a decrease in freezing during fear conditioning training (0.5, 2.8 ± 1.0%; 1.5, 2.4 ± 0.8%; 2.5, 7.6 ± 1.9%; 3.5, 18.2 ± 2.7%; 4.5, 28.4 ± 3.5%; 5.5, 30.5 ± 3.9%) compared with controls (0.5, 1.8 ± 0.4%; 1.5, 3.1 ± 0.6%; 2.5, 11.8 ± 2.4%; 3.5, 32.0 ± 3.7%; 4.5, 45.7 ± 3.5%; 5.5, 58.9 ± 3.3%; Fig. 8*E*; treatment, *F*_(1,34)_ = 18.29; *p* = 0.0001; mixed-effect analysis; *N* = 16–18 mice per experimental group). AAV-DTA mice also exhibit decreased freezing during contextual fear recall (3.5, 21.1 ± 3.3%; 4.5, 24.3 ± 3.5%) and cued fear recall (2.5, 22.6 ± 2.8%; 4.5, 16.5 ± 1.9%) compared with controls (contextual: 3.5, 46.0 ± 4.5%; 4.5, 50.4 ± 5.2%; cued: 2.5, 37.7 ± 4.5%; 4.5, 42.0 ± 5.7%; Fig. 8*F*; treatment, *F*_(1,34)_ = 15.08; *p* = 0.0005; mixed-effect analysis; Fig. 7*G*, treatment, *F*_(1,34)_ = 10.49; *p* = 0.003; mixed-effect analysis; *N* = 17–18 mice per experimental group). These data demonstrate that the loss of PV interneurons in the BLA is sufficient to impair fear learning and recall of fear memories.

## Discussion

There is a need to better understand the mechanisms contributing to the well-established bidirectional pathophysiological relationship between psychiatric illnesses and epilepsy. Our laboratory has demonstrated the critical role of PV interneurons in driving BLA network states that govern behavioral states (Antonoudiou et al., 2022). Therefore, this study focused on whether the loss of PV interneurons in the BLA may contribute to behavioral deficits associated with epilepsy. Here we demonstrate profound behavioral deficits in chronically epileptic male C57Bl6/J mice (Fig. 1) associated with a loss and dysfunction of PV interneurons in the BLA (Figs. 2, 3). Chronically epileptic mice also exhibit altered BLA network states (Fig. 4) associated with the behavioral deficits. In an effort to directly determine whether deficits in PV interneurons in the BLA contributes to the network and behavioral deficits associated with chronic epilepsy, we used an AAV-Flex-DTA approach in PV-Cre mice to ablate an equivalent number of PV interneurons in the BLA to that observed in chronic epilepsy (Fig. 5). We demonstrate that the loss of PV interneurons in the BLA is sufficient to induce behavioral deficits in fear learning and contextual and cued fear recall (Fig. 8). These data demonstrate that the loss of PV interneurons in the BLA is capable of altering behavioral states, consistent with the critical role that PV interneurons in the BLA play orchestrating network states related to fear learning (Davis et al., 2017; Ozawa et al., 2020; Antonoudiou et al., 2022). However, our data demonstrate that mice exhibiting a more severe behavioral phenotype have a consistent and profound ablation of PV interneurons in the BLA; however, the extent of PV interneuron ablation is not significantly different than those with a modest behavioral phenotype. Thus, it is likely that this pathophysiological mechanism may contribute to behavioral deficits associated with chronic epilepsy, but there are likely additional pathophysiological mechanisms at play. For example, there is a loss of interneurons in the BLA associated with epilepsy (Callahan et al., 1991; Tuunanen et al., 1996; Pitkanen et al., 1998; nicely reviewed in Aroniadou-Anderjaska et al., 2008), including interneuron subtypes other than PV interneurons, such as SST interneurons (Sperk et al., 1986; Tuunanen et al., 1997; Pitkanen et al., 1998). Thus, it is likely the loss of other interneuron subtypes may also contribute to the network and behavioral changes observed in chronically epileptic mice. The current study focused solely on PV interneurons based on the well-established role of this interneuron class in generating oscillations in the BLA (Antonoudiou et al., 2022), but cannot rule out contributions from other interneuron subtypes. Furthermore, this study looked at the number of PV interneurons and did not examine the number of synaptic contacts, which may contribute to the differences between the PV interneuron loss and functional changes in inhibitory signaling in the BLA.

The loss of PV interneurons in the BLA also does not completely recapitulate the network and behavioral changes associated with chronic epilepsy. PV interneuron ablation in the BLA results in similar avoidance behaviors in the open field and light/dark box; however, the avoidance behaviors in the elevated plus maze are not as pronounced as observed in chronically epileptic mice. This difference appears to be due to a reduced number of open arm entries in the control mice in the DTA cohort. In fact, the number of entries into the open arm of the elevated plus maze is similar for mice with PV interneuron ablation and chronically epileptic mice. Other differences between these cohorts include reduced anhedonia and more robust deficits in fear learning in mice with PV interneuron ablation in the BLA compared with chronically epileptic mice. These differences are not surprising given that there are other brain regions which are impacted in chronically epileptic animals, the most obvious of which is the hippocampus. For example, the CA2 region of the hippocampus has been shown to contribute to seizure activity in chronically epileptic mice (Whitebirch et al., 2022;Lisgaras et al., 2023) and, given the role in social behaviors and memory (Hitti and Siegelbaum, 2014), may contribute to some of the observed behavioral changes in the vIHKA model. There is well-documented interneuron loss in the hippocampus (Liu et al., 2014) which could also influence behavioral outcomes in this model. It is likely that in the AAV-DTA model, the remaining PV interneurons in the BLA may be fully functional and able to compensate for the loss of PV interneurons, whereas, in the chronic epilepsy model, the remaining PV interneurons are dysfunctional (Fig. 3). Importantly, other interneuron subtypes in the BLA may also be impacted by epilepsy, but not AAV-DTA ablation. Furthermore, while we demonstrate that AAV-DTA ablation impact PV interneuron function, a limitation of the current study is that we did not directly evaluate the impact on principal neuron function, although this may be inferred from differences in BLA network and behavioral states. Finally, AAV expression has been shown to induce glial dysfunction which was not explored in the current study and may impact synaptic transmission and neuronal excitability (Ortinski et al., 2010). These data demonstrate that ablating PV interneurons in the BLA, even more than that observed in chronically epileptic mice, does not fully recapitulate the neuropathological features or impact on network and behavioral states to the same degree that is observed in chronic epilepsy. Thus, it is likely that the loss of PV interneurons in the BLA only partially contributes to the behavioral comorbidities associated with chronic epilepsy.

The current study focused on interneuron dysfunction given our existing knowledge regarding the role of interneurons in the BLA in driving behavioral states (Likhtik et al., 2014; Stujenske et al., 2014; Felix-Ortiz et al., 2016; Davis et al., 2017; Ozawa et al., 2020; for review see Tovote et al., 2015; Antonoudiou et al., 2022). However, the data presented here also speak to changes in excitatory synaptic inhibition. There is a shift in the cumulative distribution of the amplitude and frequency of sEPSCs on PV interneurons in the BLA in chronically epileptic mice (Fig. 3). Additionally, there is an increase in the amplitude of sEPSCs and a shift in the cumulative distribution of the IEI of sEPSCs on principal neurons in the BLA (Extended Data Fig. 3-1). The current study did not examine potential changes in synaptic contacts which may accompany the observed functional changes. Surgical procedures and/or AAV expression may also introduce neuronal or glial dysfunction which could influence synaptic physiology, seizure susceptibility, and consequently behavioral abnormalities (Ortinski et al., 2010). Thus, it is clear that there are microcircuit changes beyond interneuron dysfunction which may contribute to the changes in network and behavioral states associated with chronic epilepsy.

Seizures are the most obvious manifestation of network dysfunction in epilepsy and, therefore, the primary focus of epilepsy research. We propose that more subtle deficits in network function may also underlie the risk for psychiatric illnesses comorbid with epilepsy. In fact, similar networks have been implicated in both epilepsy and psychiatric illnesses (Gilliam et al., 2004; Yang et al., 2018). Consistent with this theory, psychiatric illnesses are more common in patients with TLE (Kanner, 2017), suggesting that dysfunction in networks implicated in mood may increase vulnerability to psychiatric comorbidities. The majority of studies on TLE focus on the hippocampus. The current study suggests that other brain regions may also be altered in TLE models and may thereby contribute to the larger symptom presentation associated with chronic epilepsy, such as psychiatric comorbidities.

The amygdala is known to play a critical role in emotional processing (LeDoux, 2000) and has been implicated in numerous psychiatric illnesses, with changes in activity correlating with symptom presentation (Li et al., 2015; He et al., 2016; Satterthwaite et al., 2016), outcome prediction (Zhou et al., 2012; Connolly et al., 2017), and treatment response (Cullen et al., 2016; Fullana et al., 2017; Ellard et al., 2018). Findings from the current study in mice suggest that the amygdala may also be involved in comorbid behavioral deficits and epilepsy. These data are supported by clinical studies demonstrating altered amygdalar structure and enlargement associated with comorbid depression and epilepsy (Briellmann et al., 2007; Lv et al., 2014) and altered functional connectivity emanating from the amygdala in patients with TLE associated with psychiatric symptoms (Doucet et al., 2013). Amygdalar volume has been correlated with dysphoric disorders in epilepsy, including emotional instability, dysphoria, irritability, and aggression (Elst et al., 2009). Emerging and accumulating evidence points to amygdalar dysfunction in contributing to the full spectrum of symptoms in patients with TLE (Kullmann, 2011).

Given the evidence that the network communication within and between brain regions, such as the mPFC and BLA, drive specific behavioral states, particularly those with potential relevance to related to fear, anxiety, and depression (Likhtik et al., 2014; Stujenske et al., 2014; Felix-Ortiz et al., 2016; Davis et al., 2017; Ozawa et al., 2020; for review, see Tovote et al., 2015; Antonoudiou et al., 2022), it is necessary to explore how this network communication may become disrupted in epilepsy. Changes in functional connectivity involving the amygdala have been associated with psychiatric comorbidities in epilepsy (D. Yilmazer-Hanke et al., 2016; Colmers and Maguire, 2020). However, few studies have explored functional changes in network communication associated with comorbid psychiatric illnesses and epilepsy. Here, we demonstrate that BLA network states are altered in chronically epileptic mice (Fig. 4). We propose that the corruption in network activity within the BLA, and potentially between brain regions, contributes to the behavioral deficits associated with chronic epilepsy in mice. Further studies are required to fully understand changes in the network communication between brain regions and the impact on behavioral states associated with epilepsy. Furthermore, the network communication driving behavioral states is dynamic (Likhtik et al., 2014; Stujenske et al., 2014; Felix-Ortiz et al., 2016; Davis et al., 2017; Ozawa et al., 2020; for review, see Tovote et al., 2015; Antonoudiou et al., 2022). The current study focused on changes in network states at the baseline and did not explore dynamic changes associated with dynamic shifts in behavioral states. Thus, additional studies will be required to fully understand the relationship between network and behavioral states under pathological conditions, such as epilepsy.

This study investigates the cellular and circuit mechanisms contributing to comorbid behavioral deficits in epilepsy, pointing to the role of PV interneuron loss in the BLA and dysfunction in the network communication driving behavioral states. Despite the well-established role of network communication between brain regions driving behavioral states, this is the first study to our knowledge investigating changes in network communication under pathological conditions in contributing to comorbid behavioral deficits. These data demonstrate that altered network states in brain regions involved in emotional processing likely contribute to comorbid behavioral changes associated with chronic epilepsy.

## Synthesis

Reviewing Editor: Harald Sontheimer, University of Virginia Decisions are customarily a result of the Reviewing Editor and the peer reviewers coming together and discussing their recommendations until a consensus is reached. When revisions are invited, a fact-based synthesis statement explaining their decision and outlining what is needed to prepare a revision will be listed below. The following reviewer(s) agreed to reveal their identity: Bhanu Tewari.

Both referees find your manuscript of interest yet further revision are necessary. Importantly referee #2 feels that the data currently don't fully support the conclusions.

In the manuscript entitled "Loss of PV interneurons in the BLA contributes to altered network and behavioral states in 1 chronically epileptic mice" the authors intend to address the role of PV neurons in BSA in chronic epilepsy-associated mood disorders. The authors used an intrahippocampal KA injection mice model of chronic seizure and performed behavioral tests, immunohistochemistry, and whole cell patch clamp/field potential electrophysiology experiments. The authors began by assessing several behavioral paradigms and the fate of PV neurons in BLA, followed by recording synaptic activity, excitability, and biophysical properties of PV and principal neurons in BSA of chronically epileptic mice. The authors observed increased avoidance behavior and loss of PV interneurons in chronically epileptic mice. At the single cell and network level, authors found an increase in excitatory currents, a decrease in inhibitory currents, and an altered resonance frequency of PV neurons. Finally, to a certain extent, authors mimic the chronic seizure-induced PV loss, change in electrophysiological properties, and behavioral abnormalities in control mice using an AAV-DTA approach.

Overall, the manuscript is interesting and addresses an important question on the role of PV neurons in epilepsy-associated mood disorders. The findings may also be relevant in a broader context of cognitive decline associated with chronic epilepsy. The experimental design, sample size, and quality of data seem robust and by and large support the claims made by authors in the manuscript. I have a few concerns that need to be addressed.

1. The entire study is based on the underlying condition of mice being chronically epileptic; however, this data comes quite later in the manuscript (Figure 5). I think it makes more sense to present OR refer to it in the beginning as readers would want to know in the beginning how authors ensured the generation of chronic seizures in these mice. I understand that authors present this data from a network dysfunction perspective and with their frequency analysis over time it is sensible, however, this data also serves as evidence of mice being chronically epileptic.

2. Did all mice show chronic recurrent seizures? I assume not all, if so, did the author use the non-epileptic, but KA-injected mice as a secondary control to assess the necessity of chronic seizures for the loss of PV neurons and behavioral impairments?

3. From the literature, it does not seem uncommon to expect a generalized neuronal loss in limbic structures in chronically epileptic mice. How selective is the loss of PV neurons in BLA in this study? Did the authors compare a ratio of excitatory and PV neurons to assess this? I think it is important because the altered synaptic and network activity would be an outcome of mixed loss of both excitatory and inhibitory neurons.

4. Also, from the IHC images in Fig. 2C and D, it is hard to discern that the area presented is BSA. Since this result is the foundation of the rest of the study, it would be convincing if the authors could present a low-power image and a magnified version of BSA from the same image and delineate BLA between medial and lateral intercalated cell mass (ITC) using PV and GAD-67 markers.

5. The electrophysiology data is stunning, however, in many figures the significance labels do not indicate the two groups that are compared in the bar diagrams with multiple comparison groups. A uniform scheme to indicate the comparative groups such as authors did for Fig. 6B (tau in control vs DTA bar graph) should be helpful in other figures too.

6. Also, in several bar/whisker plots (Figs. 2B, 3H-I, 4H-I, etc.) the individual data points are missing.

7. A few limitations that need to be explicitly stated in the manuscript include, the authors did not assess(or present data) the fate of synaptic contacts in BSA using IHC to further substantiate the changes in synaptic physiology; AAV may introduce glial dysfunction that can contribute to seizure activity consequently trigger behavioral abnormality (PMID: 20418874); abnormal social behavior may be attributed to the IHKA injection causing neuronal loss in hippocampal CA2 (PMID: 24572357).

In this the manuscript the authors aim to uncover the cellular determinants of behavioral impairment that is concurrent with epilepsy. They use intrahippocampal kainic acid to generate a TLE mouse. In this model they show 1) a reduction in PV neurons of the BLA; 2) altered synaptic signaling in both the remaining PV and principal neurons; 3) decreased EEG power in the BLA in vivo; and 4) increased avoidance behavior and decreased anhedonia in this model. To mimic the loss of the PV neurons in absence of TLE, the authors injected DTA expressing vectors into the BLA. This caused a similar reduction in PV neurons of the BLA compared to the TLE mice. They then found that the remaining PV neurons were hypoexcitable and fear conditioning, but not avoidance behaviors, were altered. Overall, the experimental results are of high quality, but the results generally do not support the main conclusions. Due to the nature of the major concerns below I recommend revisions be made to validate the conclusions and improve the manuscript.

Major concerns

• To be causal, the two experiments should be combined. Use DTA to eliminate PV neurons in the BLA and then do IHKA in half and IHSa in the other half of the mice. Compare the behavioral responses of both groups to see if there are differences. Otherwise, at best this is a possible mechanism, and should be stated as such in the title, conclusion, etc.

• What is most concerning, is that there is no overlap in behavioral test results between IHKA and DTA experiments. Only avoidance tests were done for both DTA and IHKA, which were suppressed in IHKA but not in DTA. This actually suggests that PV neurons in the BLA are not involved in the behavioral effects of chronic epilepsy, the opposite of the title of the paper. Testing other experiments may provide common results between the two paradigms:

• Assess fear conditioning in IHKA mice.

• Assess sucrose test in DTA mice.

• Even if the above are satisfied, more should be done to convince of the experimental design.

o More data should be provided concerning the selectivity of neuronal loss.

• IHKA and DTA: Assess loss of other neurons in BLA - i.e. principal neurons with NeuN and SST+ neurons, for example. There is also the possibility of astrogliosis in both models. As an aside, combined PV/SST expression of DTA might provide a larger effect on the behavioral tests.

• IHKA and DTA experiments: Assess PV neuron loss in areas immediately adjacent to BLA.

o In addition, the effect of DTA on PSCs of PV neurons and all principal neuron electrophysiology was left out. This would be necessary for a full comparison of the two models and could explain the lack of effects seen in behavioral tests.

Minor concerns

Methods

• More details on the IHKA model. Do the mice go into status? Are they then rescued? Did any mice die in this procedure? Did any not develop seizures and were left out?

• Was the assessment of PV in the BLA and DTA injection bilateral or unilateral?

• How many mice were used in total? Did each mouse receive all behavioral tests and seizure monitoring? Or were there subsets for each experiment.

Fig 3

• 3a - is the significance level **** correct for IEI? Visually there is a lot of overlap compared to other cumulative distribution plots with higher p values.

Fig 3-1

• In A (right) and B (left) a highly significant difference in the cumulative probabilities is observed with a nonsignificant difference in the mean values. What does this mean? Why show both CP and means?

Fig4

• legend says "... development of SE" Shouldn't it say, "development of TLE"?

• It would be nice to have pre ihka injection power levels.

• Injection of KA lowers power levels at week 1. All other differences are due to this, not an actual increase compared to control. Comparing the differences is a bit misleading.

• 4M is out of order in text. Also, it doesn't seem to line up with data in F, which is where it should come from.

Fig 7,7-1

• In the results (lines 524-526), it says in vivo and ex vivo decrease in ability to coordinate gamma oscillation but no differences found in vivo? Perhaps that figure is just difficult to follow. Since the main point is to compare the difference between DTA and control, a figure that actually depicts this for the restraint stress could help.

General

• It could be nice to correlate responses with seizure severity, although I understand if that is not always recorded for each animal.

• It is not clear

## Author Response

The authors would like to sincerely thank the reviewers for their positive review of the study and concrete steps to improve the manuscript. We are encouraged that the Reviewer's find the manuscript to be "interesting" and "addresses and important question". Further, we are pleased that the Reviewer's find the data to be "robust", "high quality", and "support the claims" in the manuscript. Based on the reviewer's comments, we have performed additional experiments, run additional analyses, reorganized the text, and clarified our interpretation of the results. Please find our point-by-point response to the Reviewers' comments below.

Reviewer 1 Overall, the manuscript is interesting and addresses an important question on the role of PV neurons in epilepsy-associated mood disorders. The findings may also be relevant in a broader context of cognitive decline associated with chronic epilepsy. The experimental design, sample size, and quality of data seem robust and by and large support the claims made by authors in the manuscript. I have a few concerns that need to be addressed. Thank you to the Reviewer for the positive feedback on the work and the constructive suggestions to improve the manuscript.

1. The entire study is based on the underlying condition of mice being chronically epileptic; however, this data comes quite later in the manuscript (Figure 5). I think it makes more sense to present OR refer to it in the beginning as readers would want to know in the beginning how authors ensured the generation of chronic seizures in these mice. I understand that authors present this data from a network dysfunction perspective and with their frequency analysis over time it is sensible, however, this data also serves as evidence of mice being chronically epileptic. The authors thank the Reviewer for this suggestion. We have now reorganized the text to verify that the mice being used in this study are chronically epileptic. This information is now included in methods section and the first paragraph of the Results section.

2. Did all mice show chronic recurrent seizures? I assume not all, if so, did the author use the non-epileptic, but KA-injected mice as a secondary control to assess the necessity of chronic seizures for the loss of PV neurons and behavioral impairments? All mice in the vIHKA group had verified spontaneous recurrent seizures. This is now stated in the Methods and Results sections. We employed mice infused with saline (vIHSa) as controls.

3. From the literature, it does not seem uncommon to expect a generalized neuronal loss in limbic structures in chronically epileptic mice. How selective is the loss of PV neurons in BLA in this study? Did the authors compare a ratio of excitatory and PV neurons to assess this? I think it is important because the altered synaptic and network activity would be an outcome of mixed loss of both excitatory and inhibitory neurons. The Reviewer is correct. There is documented loss of GAD67+ neurons and SST interneurons in the BLA in chronically epileptic mice (Pitkanen, 1998), which we reference in the manuscript. In an effort not to perform redundant studies and due to our focus on BLA network states, we focused on PV interneurons in the current study based on our previous studies demonstrating a critical role for PV interneurons in orchestrating oscillations in the BLA (Antonoudiou, 2023). However, in response to the Reviewer's comments, we do acknowledge that deficits in other interneuron subtypes in chronically epileptic mice may also contribute to the observed deficits in network and behavioral states in chronically epileptic mice and justified the focus of the current study on PV interneurons. This is addressed in both the Introduction and Discussion sections. We performed additional experiments to look at the expression of NeuN in the BLA. We do not observe any change in NeuN expression in the BLA, suggesting that there are is not a substantial loss of principal neurons, the predominant neuronal cell type, in the BLA in chronically epileptic mice. This data is now included in the manuscript (Figure 2).

4. Also, from the IHC images in Fig. 2C and D, it is hard to discern that the area presented is BSA. Since this result is the foundation of the rest of the study, it would be convincing if the authors could present a low-power image and a magnified version of BSA from the same image and delineate BLA between medial and lateral intercalated cell mass (ITC) using PV and GAD-67 markers. Lower and high magnification images are now provided for Figure 2.

5. The electrophysiology data is stunning, however, in many figures the significance labels do not indicate the two groups that are compared in the bar diagrams with multiple comparison groups. A uniform scheme to indicate the comparative groups such as authors did for Fig. 6B (tau in control vs DTA bar graph) should be helpful in other figures too. Thank you to the Reviewer for this suggestion. The figures now include a uniform scheme to indicate comparisons and statistical significance.

6. Also, in several bar/whisker plots (Figs. 2B, 3H-I, 4H-I, etc.) the individual data points are missing. We have now included individual datapoints.

7. A few limitations that need to be explicitly stated in the manuscript include, the authors did not assess(or present data) the fate of synaptic contacts in BSA using IHC to further substantiate the changes in synaptic physiology; AAV may introduce glial dysfunction that can contribute to seizure activity consequently trigger behavioral abnormality (PMID: 20418874); abnormal social behavior may be attributed to the IHKA injection causing neuronal loss in hippocampal CA2 (PMID: 24572357). We have now added these topics and citations to the discussion.

Reviewer 2 In this the manuscript the authors aim to uncover the cellular determinants of behavioral impairment that is concurrent with epilepsy. They use intrahippocampal kainic acid to generate a TLE mouse. In this model they show 1) a reduction in PV neurons of the BLA; 2) altered synaptic signaling in both the remaining PV and principal neurons; 3) decreased EEG power in the BLA in vivo; and 4) increased avoidance behavior and decreased anhedonia in this model. To mimic the loss of the PV neurons in absence of TLE, the authors injected DTA expressing vectors into the BLA. This caused a similar reduction in PV neurons of the BLA compared to the TLE mice. They then found that the remaining PV neurons were hypoexcitable and fear conditioning, but not avoidance behaviors, were altered. Overall, the experimental results are of high quality, but the results generally do not support the main conclusions. Due to the nature of the major concerns below I recommend revisions be made to validate the conclusions and improve the manuscript. The authors thank the Reviewer for the constructive feedback to improve the manuscript.

Major concerns • To be causal, the two experiments should be combined. Use DTA to eliminate PV neurons in the BLA and then do IHKA in half and IHSa in the other half of the mice. Compare the behavioral responses of both groups to see if there are differences. Otherwise, at best this is a possible mechanism, and should be stated as such in the title, conclusion, etc. This is an important distinction. The title has been changed to "Loss of PV interneurons in the BLA may contribute to altered network and behavioral states in chronically epileptic mice" and edits have been made throughout the text to temper this conclusion. • What is most concerning, is that there is no overlap in behavioral test results between IHKA and DTA experiments. Only avoidance tests were done for both DTA and IHKA, which were suppressed in IHKA but not in DTA. This actually suggests that PV neurons in the BLA are not involved in the behavioral effects of chronic epilepsy, the opposite of the title of the paper. Testing other experiments may provide common results between the two paradigms: We have now included additional behavioral tests for consistency between the IHKA and DTA groups. • More data should be provided concerning the selectivity of neuronal loss. We have now provided additional data examining NeuN expression in the BLA of vIHKA mice. We have also verified that there is no loss of other interneuron subtypes, such as SST interneurons, in the AAV-DTA model. These data are now provided in the manuscript. • IHKA and DTA: Assess loss of other neurons in BLA - i.e. principal neurons with NeuN and SST+ neurons, for example. There is also the possibility of astrogliosis in both models. As an aside, combined PV/SST expression of DTA might provide a larger effect on the behavioral tests. We have now provided additional data examining NeuN expression in the BLA of vIHKA mice. We have also verified that there is no loss of other interneuron subtypes, such as SST interneurons, in the AAV-DTA model. These data are now provided in the manuscript. • IHKA and DTA experiments: Assess PV neuron loss in areas immediately adjacent to BLA. While we were not able to quantify PV interneuron expression in other brain regions, we do provide a representative image of the targeting which demonstrates spatially restricted manipulations in the BLA (Figure 5E). • In addition, the effect of DTA on PSCs of PV neurons and all principal neuron electrophysiology was left out. This would be necessary for a full comparison of the two models and could explain the lack of effects seen in behavioral tests. We did not assess the impact of the DTA manipulation on the properties of principal BLA neurons, rather we focused our assessments on the impact of this manipulation on the expression and function of PV interneurons to verify that the AAV-DTA manipulation is inducing the expected changes. Since we did not assess the impact of the AAV-DTA manipulation on principal neurons, we acknowledge this limitation and interpret the results in light of this limitation which is discussed in the Discussion section.

Minor concerns Methods • More details on the IHKA model. Do the mice go into status? Are they then rescued? Did any mice die in this procedure? Did any not develop seizures and were left out? More information is now provided regarding the vIHKA model. All mice in the vIHKA group had verified spontaneous recurrent seizures. This is now stated in the Methods and Results sections. We employed mice infused with saline (vIHSa) as controls. Was the assessment of PV in the BLA and DTA injection bilateral or unilateral? More information is now provided about the quantification of PV interneuron loss in the BLA of the vIHKA and DTA models. • How many mice were used in total? Did each mouse receive all behavioral tests and seizure monitoring? Or were there subsets for each experiment. The same mice were not used across all experiments and, thus, unfortunately, we cannot make correlations across these outcome measures. The numbers of mice is provided for each experiment.

Fig 3 • 3a - is the significance level **** correct for IEI? Visually there is a lot of overlap compared to other cumulative distribution plots with higher p values. We corrected this in the text.

Fig 3-1 • In A (right) and B (left) a highly significant difference in the cumulative probabilities is observed with a nonsignificant difference in the mean values. What does this mean? Why show both CP and means? We have clarified this in the text.

Fig4 • legend says "... development of SE" Shouldn't it say, "development of TLE"? Thank you for this important catch. We have altered the legend to state TLE rather than SE. • It would be nice to have pre ihka injection power levels. Technical limitations prevent us from making this comparison since mice are implanted with electrodes at the time of the vIHKA surgery. • Injection of KA lowers power levels at week 1. All other differences are due to this, not an actual increase compared to control. Comparing the differences is a bit misleading. We agree with this concern and the data is now represented as % total power rather than normalized to Week 1. • 4M is out of order in text. Also, it doesn't seem to line up with data in F, which is where it should come from. We have reordered the reference to the figures in the text and verified that the information in Figure 4M is correct.

Fig 7,7-1 • In the results (lines 524-526), it says in vivo and ex vivo decrease in ability to coordinate gamma oscillation but no differences found in vivo? Perhaps that figure is just difficult to follow. Since the main point is to compare the difference between DTA and control, a figure that actually depicts this for the restraint stress could help. The authors thank the Reviewer for pointing out that the explanation and presentation of this data was confusing. We have now removed the ex vivo data from the manuscript and focus solely on the in vivo data.

General • It could be nice to correlate responses with seizure severity, although I understand if that is not always recorded for each animal. This is an excellent suggestion but unfortunately, we do not have the seizure data in enough animals to make that correlation. However, we agree that this would be incredibly informative and we will plan to do this in the future.
