# Supplementary figures and images for: Loss of PV Interneurons in the BLA May Contribute to Altered Network and Behavioral States in Chronically Epileptic Mice
Source: eNeuro. 2025 Jan 16;12(1):ENEURO.0482-23.2024. doi: 10.1523/ENEURO.0482-23.2024 (PMC11773627; doi:10.1523/ENEURO.0482-23.2024)

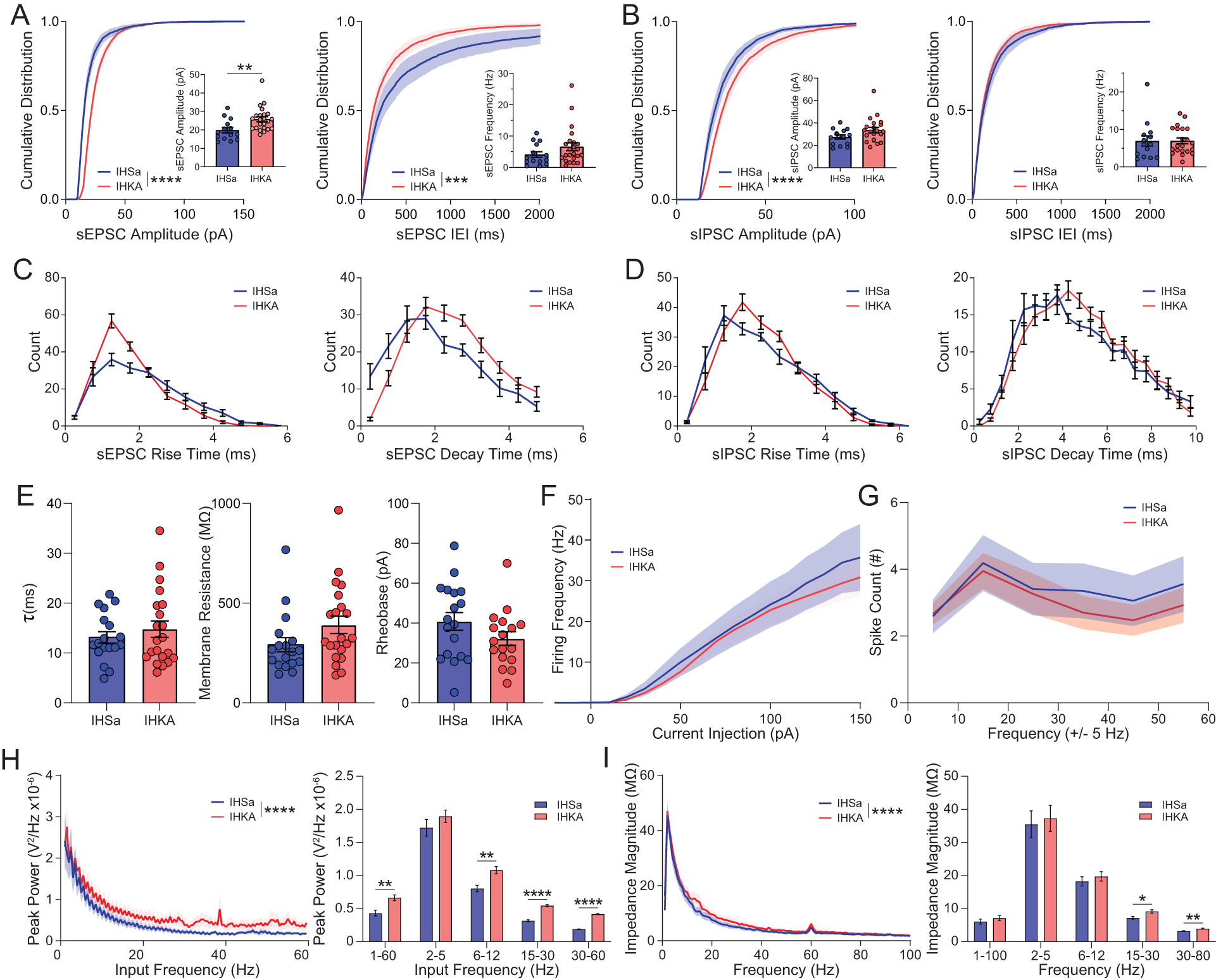

Supplement: Figure 3-1 — Active and passive electrophysiological properties of BLA Principal neurons. A. Whole-cell patch clamp recordings were taken from BLA principal neurons, and the cumulative distribution of sEPSCs in IHKA mice were observed to have a right-shift in amplitude and a left-shift in IEI, with a corresponding increase in mean amplitude, but no significant difference in mean frequency. B. BLA principal neurons showed a right-shift in the cumulative distribution of sIPSC amplitude in IHKA mice with no change in sIPSC IEI, with no significant changes in the mean amplitude or frequency of sIPSC afferents. Both sEPSCs and sIPSCs saw no significant changes in rise (C) or decay (D) kinetics between IHSa and IHKA mice. E, Intrinsic membrane properties including the membrane time constant (left), membrane resistance (middle), and rheobase (right) are not significantly altered in BLA principal neurons in IHKA mice. Firing properties including the input-output relationship (F) and firing in response to a supra-threshold chirp stimulation (G) did not reveal any significant differences between IHSa and IHKA mice. H, A subthreshold Chirp current (top) was injected into the current-clamped principal neuron to determine intrinsic passive resonant membrane properties at different input frequencies, IHKA principal neurons exhibited increased resonant properties across the wavelengths tested (left) and within specific high-frequency bands (right). I, Membrane impedance as a function of frequency was not significantly higher in IHKA across the entire frequency range (left) however there were some higher frequency bands that were significantly increased (right). * denotes the degree of significance between conditions. Cumulative distributions in A, B derive significance from Kolmogorov-Smirnov tests. The mean comparisons in A, B, and E derive significance from unpaired 2-tailed t-tests. Histograms in C, D derive significance from Wilcoxon matched pairs signed rank test, 2-tailed. The two-factor [file eneuro-12-ENEURO.0482-23.2024-s002.tif]
